# *CYTOR* Facilitates Formation of FOSL1 Phase Separation and Super Enhancers to Drive Metastasis of Tumor Budding Cells in Head and Neck Squamous Cell Carcinoma

**DOI:** 10.1002/advs.202305002

**Published:** 2023-11-30

**Authors:** Wenjin Wang, Bokai Yun, Rosalie G Hoyle, Zhikun Ma, Shadid Uz Zaman, Gan Xiong, Chen Yi, Nan Xie, Ming Zhang, Xiqiang Liu, Dipankar Bandyopadhyay, Jiong Li, Cheng Wang

**Affiliations:** ^1^ Hospital of Stomatology Sun Yat‐sen University Guangzhou 510055 China; ^2^ Guangdong Provincial Key Laboratory of Stomatology Guangzhou 510080 China; ^3^ Guanghua School of Stomatology Sun Yat‐sen University Guangzhou 510055 China; ^4^ Department of Medicinal Chemistry School of Pharmacy Virginia Commonwealth University Richmond VA 23298‐0540 USA; ^5^ Department of Oral and Maxillofacial Surgery Nanfang Hospital, Southern Medical University Guangzhou 510515 China; ^6^ Department of Biostatistics School of Medicine Virginia Commonwealth University Richmond VA 23298‐0540 USA; ^7^ Massey Cancer Center Virginia Commonwealth University Richmond VA 23298‐0540 USA; ^8^ Department of Oral and Craniofacial Molecular Biology School of Dentistry Virginia Commonwealth University Richmond VA 23298‐0540 USA; ^9^ Philips Institute for Oral Health Research School of Dentistry Virginia Commonwealth University Richmond VA 23298‐0540 USA

**Keywords:** CYTOR, FOSL1, head and neck squamous cell carcinoma, phase separation, super‐enhancer

## Abstract

Tumor budding (TB) is a small tumor cell cluster with highly aggressive behavior located ahead of the invasive tumor front. However, the molecular and biological characteristics of TB and the regulatory mechanisms governing TB phenotypes remain unclear. This study reveals that TB exhibits a particular dynamic gene signature with stemness and partial epithelial‐mesenchymal transition (p‐EMT). Importantly, nuclear expression of *CYTOR* is identified to be the key regulator governing stemness and the p‐EMT phenotype of TB cells, and targeting *CYTOR* significantly inhibits TB formation, tumor growth and lymph node metastasis in head and neck squamous cell carcinoma (HNSCC). Mechanistically, *CYTOR* promotes tumorigenicity and metastasis of TB cells by facilitating the formation of FOSL1 phase‐separated condensates to establish FOSL1‐dependent super enhancers (SEs). Depletion of *CYTOR* leads to the disruption of FOSL1‐dependent SEs, which results in the inactivation of cancer stemness and pro‐metastatic genes. In turn, activation of FOSL1 promotes the transcription of *CYTOR*. These findings indicate that *CYTOR* is a super‐lncRNA that controls the stemness and metastasis of TB cells through facilitating the formation of FOSL1 phase separation and SEs, which may be an attractive target for therapeutic interventions in HNSCC.

## Graphical abstract

In this article, we show that tumor budding (TB) exhibits a dynamic gene signature with stemness and partial epithelial‐mesenchymal transition (p‐EMT). Nuclear expression of *CYTOR* promotes tumorigenicity and metastasis of TB cells by facilitating the formation of FOSL1 phase‐separated condensates to establish FOSL1‐dependent super enhancers.

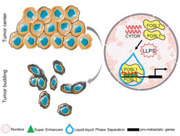

## 1 Introduction

Tumor budding (TB) is defined as the presence of isolated single cancer cells or clusters of up to four cancer cells, which are resided ahead of the invasive tumor front.^[^ ^1^ ^]^ Morphologically, TB is detached from the tumor bulk and characterized with dedifferentiation and loss of cell polarity. Currently, accumulating evidence suggests that TB is a promising prognostic biomarker in many solid cancers, which is proposed to be a source of disseminating tumor cells.^[^ ^1^, ^2^ ^]^ Biologically, TB has been hypothesized to be associated with epithelial‐mesenchymal transition (EMT) or partial EMT (p‐EMT), which is a dynamic and reversible cellular process of transdifferentiation and has a pivotal role during metastatic cascades in cancer.^[^ ^3^ ^]^ Of note, Jensen et al.^[^ ^4^ ^]^ and De Smedt et al.^[^ ^5^ ^]^ revealed the molecular signature of TB cells and highlighted the notion that TB cells undergo EMT. However, these TB signatures were only investigated in TB cells as compared with the corresponding tumor bulk cells and no corresponding normal control epithelial cells nor disseminated cancer cells were enrolled, which limits our understanding of the dynamics and plasticity of TB in the process of cancer progression.

Herein, we aimed to investigate the dynamic molecular signature of TB during metastatic cascades and clarify the underlying mechanisms governing the TB phenotype in head and neck squamous cell carcinoma (HNSCC). We revealed that 5 significant dynamic transcript expression patterns were observed in the metastatic cascades of HNSCC (clusters 0, 2, 12, 13, 14). Of note, transcripts in cluster 14 were correlated with invasive signatures and displayed an increased trend with a peak point in TB but decreased in lymph node metastasis, suggesting that cluster 14 is the specific molecular signature of TB in HNSCC. Then, the cytoskeleton regulator RNA (*CYTOR*), a long intergenic non‐coding RNA, was identified to be predominantly expressed in the nucleus of TB cells, which was correlated with malignant progression of HNSCC. Functional studies revealed that *CYTOR* promoted stemness, migration, invasion and metastasis of HNSCC in vitro and in vivo. Mechanically, we uncovered that *CYTOR* exerted its function in promoting tumorigenicity and metastasis of HNSCC through facilitating the formation of FOSL1 phase separation condensates and activating FOSL1‐dependent super enhancers (SEs). We further demonstrated that targeting *CYTOR* profoundly suppressed tumorigenesis and metastasis of HNSCC. Taken together, our results suggest that *CYTOR* is a master regulator of TB and targeting *CYTOR* can significantly inhibit the metastatic ability of HNSCC.

## 2 Results

### 2.1 Tumor Budding is a Subset of Cancer Cells with p‐EMT in HNSCC

**Figure 1:**
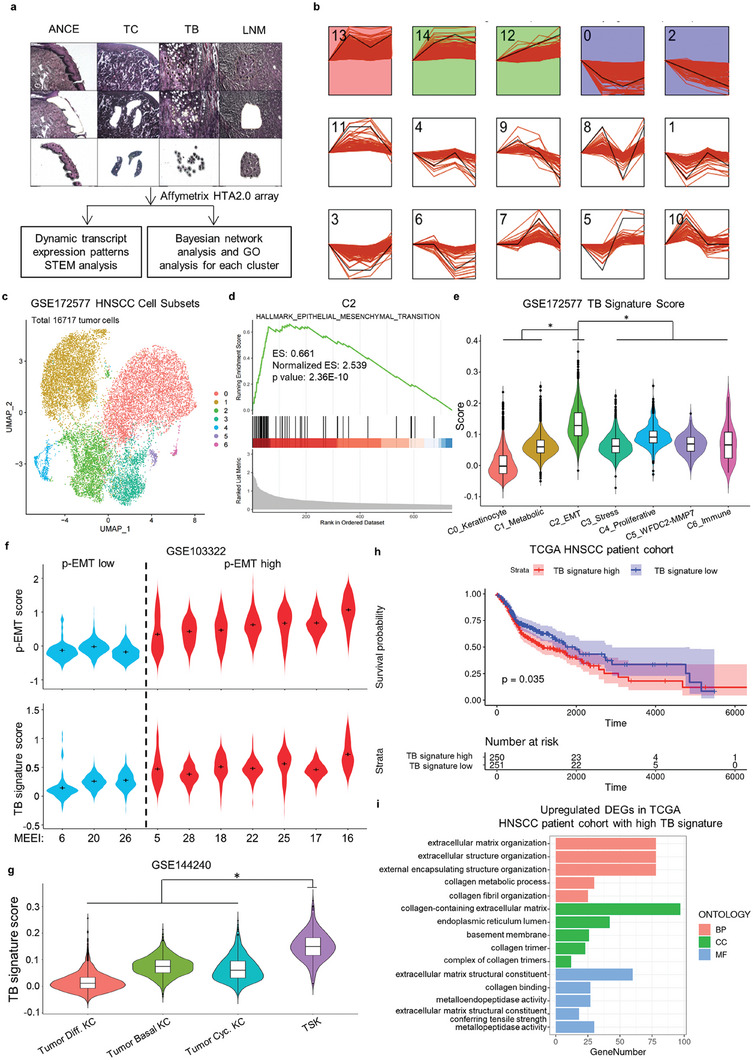
Tumor budding is a subset of cancer cells with p‐EMT in HNSCC. a) Collection of adjacent non‐cancerous epithelium (ANCE), tumor center cells (TC) and TB cells from primary HNSCC, and metastatic HNSCC cells from lymph nodes (LNM) with laser capture microdissection, and transcripts profiling analysis with HTA2.0 array. b) Different dynamic transcript expression patterns during HNSCC progressive cascades with STEM analysis. c) UMAP plots of HNSCC cells clusters colored by unique genes expression based on scRNA‐seq data of GSE172577. UMAP, Uniform Manifold Approximation and Projection. d) GSEA revealed that cluster 2 (C2) HNSCC cells display EMT signature from GSE172577. e) Violin plots of the TB signature scores in different HNSCC cells clusters from GSE172577, revealing C2_EMT displaying the highest TB signature. *p < 2.2 × 10^−16^ by pairwise Wilcoxon rank‐sum tests. f) p‐EMT scores depicted by the top panel showing different cohorts of p‐EMT high (red) and low (blue) tumors based on scRNA‐seq data of GSE103322 database. TB signature scores depicted by the bottom panel revealing p‐EMT high tumors showing high TB signature scores from GSE103322. g) Violin plots of the TB signature scores in distinct tumor subpopulation based on scRNA‐seq data of GSE144240, revealing TSK subpopulation displaying a TB signature. *p < 2.2 × 10^−16^ by pairwise Wilcoxon rank‐sum tests. h) Kaplan‐Meier curves and log‐rank test for overall survival of HNSCC patients grouped by TB signature gene expression from TCGA cohort. i) GO analysis of upregulated genes in HNSCC tissues showing high TB signature based on TCGA database.

Previously, we showed that the TB represented a subcluster of cancer cells with higher aggressive behaviors than cells located at the tumor center in HNSCC,^[^ ^2^, ^6^ ^]^ supporting the notion that TB cells might detach from the tumor bulk and then spread to cervical lymph nodes. To comprehensively dissect the molecular mechanisms governing TB cells during the progression of HNSCC, we collected adjacent non‐cancerous epithelium (ANCE), tumor center cells (TC) and TB cells from primary HNSCC, and metastatic HNSCC cells from lymph nodes (LNM) using laser capture microdissection. Then, Affymetrix HTA2.0 array was used to profile the landscape of transcripts in these cells, and the dynamic transcript expression patterns were investigated using Short Time‐series Expression Miner (STEM) analysis (**Figure** **1**a). As shown in Figure 1b and Figure S1a‐e (Supporting Information), we revealed 15 different dynamic transcript expression patterns during the progressive cascades of HNSCC, but only 5 significant dynamic transcript expression patterns were observed (cluster 0, 2, 12, 13, 14). Cluster 0 included 479 transcripts, which showed a decreased trend with a bottom point at TB. Gene Ontology (GO) analysis indicated that cluster 0 was correlated with metabolism‐related process (Figure S1a, Supporting Information). Cluster 2 included 144 transcripts and displayed a consistent decreased trend during the progressive cascades of HNSCC, which were mainly associated with peptide cross‐linking, keratinocyte differentiation, and epidermis development (Figure S1b, Supporting Information). Cluster 12 contained 294 transcripts and demonstrated a consistent increased trend, which was correlated with immune response (Figure S1c, Supporting Information). Cluster 13 was comprised of 890 transcripts and showed an increased trend with a bottom point at TB, involved in the antigen presentation and extracellular matrix organization (Figure S1d, Supporting Information). Cluster 14 included 460 transcripts and displayed an increased trend with a peak point at TB. GO analysis showed that extracellular matrix organization, extracellular matrix disassembly and cell differentiation were involved in cluster 14, supporting the invasive behavior and defined as the TB signature (Figure S1e, Supporting Information). Increasing evidence implicates that TB cells are correlated with dedifferentiation and EMT.^[^ ^1^ ^]^ Importantly, EMT and its reverse mesenchymal‐to‐epithelial transition (MET) have been evidenced to play a pivotal role in the metastatic cascades of cancers.^[^ ^7^ ^]^ Our studies indicate that TB signature genes in cluster 14 are increased in TB cells but decreased in metastatic cancers, which is a perfect match with the EMT and MET model in cancers. To further characterize whether the TB phenotype represents EMT in HNSCC, we determined the TB score in different subpopulation SCC cells based on three different scRNA‐seq datasets by using the TB signature genes. Notably, we observed that p‐EMT HNSCC cells and tumor‐specific keratinocytes (TSKs, a subset cells with high EMT score) displayed the highest TB signature (Figure 1c–g). Moreover, the TB signature indicated a poor survival rate in HNSCC from The Cancer Genome Atlas (TCGA) dataset (Figure 1h). Consistent with the GO analysis in cluster 14 (TB signature), the upregulated genes in HNSCC patients with high TB signature were also enriched in extracellular matrix organization and extracellular structure organization (Figure 1i).

### 2.2 Increase of *CYTOR* Promotes Invasion and Tumorigenicity of Tumor Budding Cells in HNSCC

**Figure 2:**
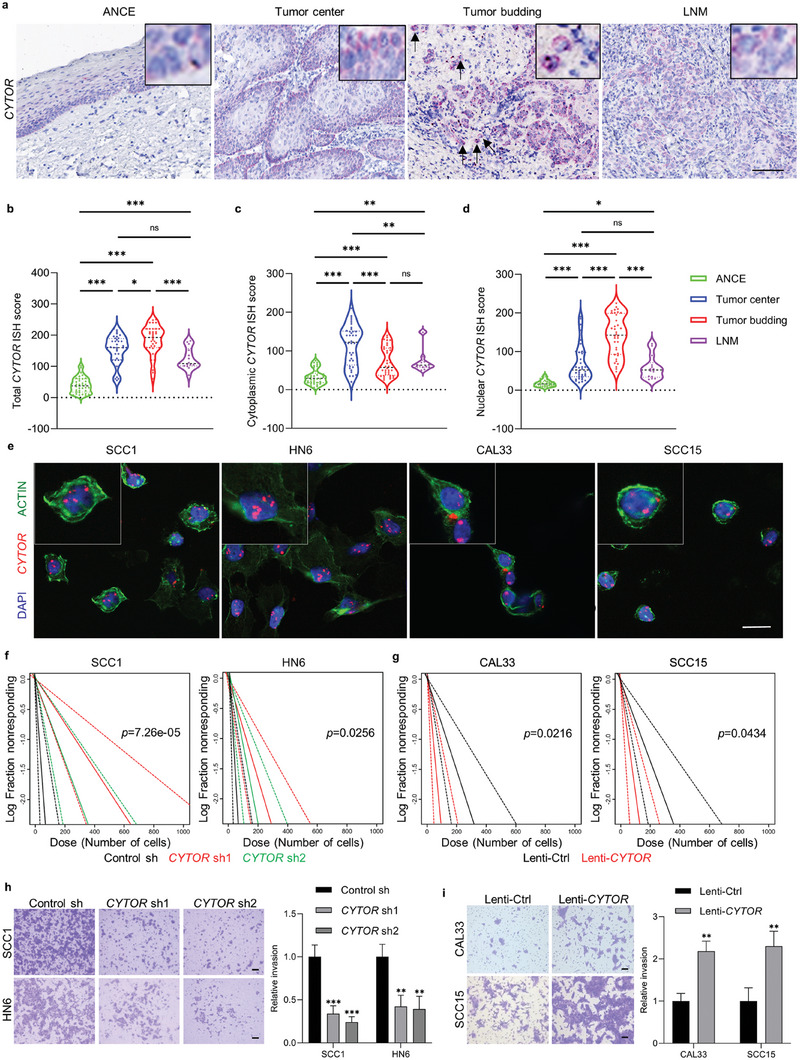
Increase of *CYTOR* promotes invasion and tumorigenicity of tumor budding cells in HNSCC. a) Representative images of *CYTOR* ISH staining in ANCE, tumor center and TB in primary HNSCC, and lymph node metastatic carcinoma (LNM). Scale bar, 100 µm. b–d) Quantification of total b), cytoplasmic c), and nuclear d) *CYTOR* expression in adjacent non‐cancerous epithelium, tumor center, TB and LNM. *p < 0.05, **p < 0.01, and ***p < 0.001 by one‐way ANOVA. ns, not significant. e) Detection of expression and subcellular localization of *CYTOR* in HNSCC cell lines SCC1, HN6, CAL33 and SCC15 by fluorescence in situ hybridization (FISH) and Immunofluorescence (IF) experiments, *CYTOR* (red) and actin (green). Scale bar, 20 µm. f,g) Evaluation of capacities of tumor sphere formation in HNSCC cells with depletion f) or overexpression g) of *CYTOR* by extreme limited dilution assay (ELDA), and the frequency and *p*‐value computed by ELDA software. h,i) Representative images and evaluation of invasion in HNSCC cells with depletion h) and overexpression i) of *CYTOR* by transwell assay. Scale bar, 200 µm. **p < 0.01 and ***p < 0.001 by one‐way ANOVA (h). **p < 0.01 by Student's *t*‐test (i).

Then, a novel Bayesian network algorithm was used to identify the key driver gene controlling TB phenotype in cluster 14. As shown in Figure S2a (Supporting Information), *CYTOR* was identified as the hottest hub lncRNA among genes in cluster 14. Of note, we observed that *CYTOR*‐related nodal genes were mainly involved in EMT, p‐EMT or tumor invasion in HNSCC, including *LAMB3*, *PDPN* and *FNDC3B*.^[^ ^8^ ^]^ These findings indicate that *CYTOR* has a critical role in modulating the behaviors of TB cells in HNSCC. Next, we evaluated the expression of *CYTOR* and investigated its correlation with HNSCC progression. RNA in situ hybridization (ISH) staining revealed that *CYTOR* was expressed in both the cytoplasm and nucleus of HNSCC cells, but mainly located at the nucleus in TB cells (**Figure** **2**a). As shown in Figure 2b–d, the ISH score of total *CYTOR* was increased dramatically in TC, TB and LNM as compared to the ANCE, which is consistent with our HTA2.0 array data. Similar results were also observed for the expression of cytoplasmic and nuclear *CYTOR*. When comparing with TC, total and nuclear *CYTOR* expression did not significantly change in LNM. Notably, TB cells had higher total and nuclear *CYTOR* expression than cancer cells in TC and/or LNM. A decreased expression of cytoplasmic *CYTOR* was observed in TB cells than in cancer cells from TC. These findings suggest that nuclear expression of *CYTOR* plays a pivotal role in regulating the aggressiveness of TB cells.

To investigate whether nuclear expression of *CYTOR* has a critical function in HNSCC cells in vitro, we first detected the expression and subcellular location of *CYTOR* in a panel of 4 HNSCC cell lines. As shown in Figure 2e and Figure S2b,c (Supporting Information), the expression of *CYTOR* is mainly abundant in the nuclei of HNSCC cell lines, especially in SCC1 and HN6 cells with high invasive capacities. To examine the functional role of *CYTOR* in governing aggressive phenotypes of HNSCC cells, lentivirus‐based *CYTOR* overexpression plasmid and two different lentivirus‐based shRNAs (*CYTOR* sh1 and *CYTOR* sh2) were generated. Real‐time quantitative PCR (RT‐qPCR) showed that ectopic expression of *CYTOR* significantly enhanced the expression of *CYTOR* in CAL33 and SCC15 cells, while both *CYTOR* sh1 and *CYTOR* sh2 were able to knock down *CYTOR* in SCC1 and HN6 cells (Figure S2d,e, Supporting Information). As shown in Figure S2f,g (Supporting Information), the ability of tumor sphere formation was significantly decreased in SCC1 and HN6 cells with depletion of *CYTOR* but enhanced in CAL33 and SCC15 cells overexpressed *CYTOR*. Of note, the extreme limited dilution assay showed that capacities of tumor sphere formation were significantly suppressed in SCC1 and HN6 cells by depletion of *CYTOR* and increased in CAL33 and SCC15 cells with overexpression of *CYTOR* (Figure 2f,g). As expected, depletion of *CYTOR* suppressed cell migration and invasion in SCC1 and HN6 cells, and overexpression of *CYTOR* did promote cell migration and invasion in CAL33 and SCC15 cells (Figure 2h,i; Figure S2h,i,Supporting Information). Taken together, these findings indicate that *CYTOR* is involved in cell migration, invasion and stemness, which is required for a metastatic phenotype of cancer cells.

### 2.3 *CYTOR* Promotes Metastasis and Tumor Growth of HNSCC In Vivo

**Figure 3:**
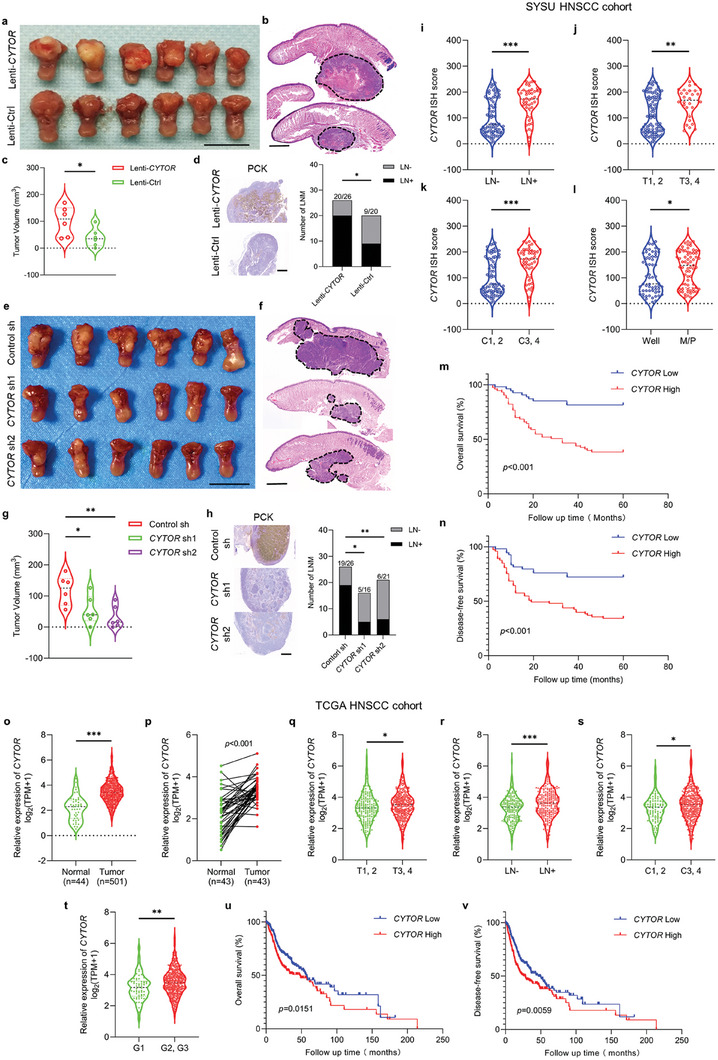
*CYTOR* promotes HNSCC tumor growth and metastasis. a) Overexpression of *CYTOR* promotes the growth of HNSCC orthotropic xenograft formed by CAL33. Scale bar, 1 cm. b) Representative images of HE staining of CAL33 formed xenograft. Scale bar, 1 mm. c) Quantification of tumor volume of HNSCC orthotropic xenograft. *p < 0.05 by Student's *t*‐test. d) Representative images of PCK IHC staining in cervical lymph node. Scale bar, 300 µm. Evaluation of the percentage of cervical lymph node with metastasis by Fisher's exact test, *p < 0.05. e) Depletion of *CYTOR* inhibits the growth of HNSCC orthotropic xenograft formed by SCC1. Scale bar, 1 cm. f) Representative images of HE staining of SCC1 formed orthotropic xenograft. Scale bar, 1 mm. g) Quantification of tumor volume of SCC1 formed orthotropic xenograft. *p < 0.05 and **p < 0.01 by one‐way ANOVA. h) Representative images of PCK IHC staining in cervical lymph node. Scale bar, 300 µm. *p < 0.05 and **p < 0.01 by Chi‐square test. i–l) Quantification of *CYTOR* expression based on ISH staining in HNSCC tissues grouped by with/without cervical lymph node metastasis i), tumor stage (T1, 2 and T3, 4) j), clinical stage (C1, 2 and C3, 4) k) and differentiation degree (well and middle plus poor) l). *p < 0.05, **p < 0.01, and ***p < 0.001 by Student's *t*‐test. m,n) Kaplan‐Meier curves for overall survival m) and disease‐free survival n) of HNSCC patients grouped by the expression of *CYTOR* based on ISH staining, and difference evaluated by log‐rank test. o) Quantification of *CYTOR* expression in normal and HNSCC tissues from TCGA database. ***p < 0.001 by Student's *t*‐test. p) Expression of *CYTOR* in 43 paired HNSCC and normal tissues from TCGA database evaluated with Paired *t*‐test. q–t) Quantification of *CYTOR* expression from TCGA database in HNSCC grouped by tumor stage (T1, 2 and T3, 4) q), with/without cervical lymph node metastasis r), clinical stage (C1, 2 and C3, 4) s) and differentiation degree (G1 and G2 plus G3) t). *p < 0.05, **p < 0.01, and ***p < 0.001 by Student's *t*‐test. u,v) Kaplan‐Meier curves and log‐rank test for overall survival u) and disease‐free survival v) of HNSCC patients grouped by the expression of *CYTOR* from TCGA database.

To determine the functional role of *CYTOR* in tumor growth and metastasis in vivo, HNSCC orthotropic xenograft models were established using CAL33 cells overexpressing *CYTOR*, SCC1 cells with depletion of *CYTOR* and their corresponding control cells were also generated. As shown in **Figure** **3**a–c, mice bearing CAL33 with overexpression of *CYTOR* showed larger tumors than mice bearing control cells. Pan‐cytokeratin (PCK) staining in cervical lymph node revealed that lymph node metastasis was significantly increased by overexpressing *CYTOR* (Figure 3d). Moreover, we also confirmed that genetic depletion of endogenous *CYTOR* inhibited tumor growth and lymph node metastasis (Figure 3e–h). These results demonstrate that *CYTOR* functions as an oncogene to promote tumor growth and metastasis of HNSCC.

To extend these findings in clinical human HNSCC samples, we further investigated correlations of *CYTOR* with clinicopathological parameters using two HNSCC patient cohorts (SYSU cohort and TCGA cohort). As shown in Figure 3i, human primary HNSCC with lymph node metastasis displayed a higher expression level of *CYTOR* than those without lymph node metastasis. Compared with T1,2 stage HNSCC patients, *CYTOR* expression was increased significantly in T3,4 stage HNSCC patients (Figure 3j). The increased expression of *CYTOR* was also observed in HNSCC patients with clinical stage III, IV as compared to those with clinical stage I, II (Figure 3k). Moreover, cancer cells with moderated or poor differentiation had higher expression of *CYTOR* than the well differentiated cells (Figure 3l). Importantly, high expression of *CYTOR* showed a decreased overall survival and disease‐free survival (Figure 3m,n). Similar results were observed in the TCGA patient cohort (Figure 3o–v). Collectively, these findings support the notion that *CYTOR* promotes metastasis and tumor growth of HNSCC, which is correlated with decreased overall survival and disease‐free survival in HNSCC patients.

### 2.4 *CYTOR* Controls FOSL1 Signaling in HNSCC

**Figure 4:**
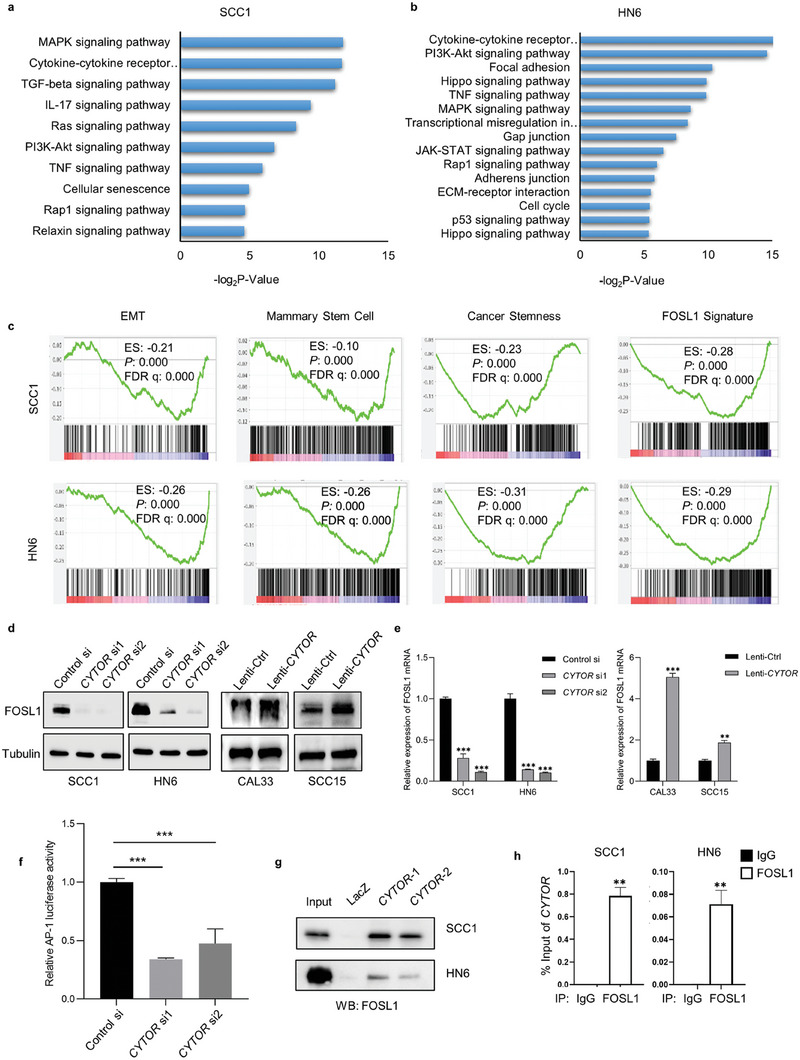
*CYTOR* controls FOSL1 signaling in HNSCC. a,b) GO analysis for *CYTOR* governing signaling pathway in SCC1 a) and HN6 b) after depletion of *CYTOR*. c) GSEA showed that the EMT, mammary stem cell, cancer stemness, and FOSL1 signature were significantly inhibited by depletion of *CYTOR* in SCC1 and HN6. d) Western‐blot analysis showed that FOSL1 protein levels were downregulated with knockdown of *CYTOR* and upregulated with overexpression of *CYTOR*. e) RT‐qPCR showed that FOSL1 transcription was suppressed by depletion of *CYTOR* in SCC1 and HN6, ***p < 0.001 by one‐way ANOVA. And FOSL1 mRNA was upregulated by overexpression of *CYTOR* in CAL33 and SCC15, **p < 0.01 and ***p < 0.001 by Student's *t*‐test. f) AP‐1 activities in HNSCC cells with/without CYTOR depletion were evaluated by luciferase reporter assay. ***p < 0.001 by one‐way ANOVA. g) RNA pull‐down and Western blot assay showed that *CYTOR* physically interacted with FOSL1 in HNSCC cells. h) RIP and RT‐qPCR showed that *CYTOR* was co‐immunoprecipitated with FOSL1. **p < 0.01 by Student's *t*‐test.

To explore the molecular mechanisms of *CYTOR* in promoting HNSCC progression, RNA‐sequencing was conducted in HNSCC cell lines, SCC1 and HN6, after transfection with *CYTOR* or control siRNAs followed by GO and Gene Set Enrichment Analysis (GSEA). As shown in **Figure** **4**a,b, several critical signaling pathways were significantly changed after *CYTOR* knockdown in HNSCC cells, including the mitogen‐activated protein kinases (MAPKs) pathway, cytokine‐cytokine receptor interactions, and PI3K‐AKT signaling. GSEA identified that the EMT, mammary stem cell, cancer stemness, and FOSL1 target gene sets’ expression levels were all significantly inhibited by knockdown of *CYTOR* in SCC1 and HN6 cells (Figure 4c). The correlation of *CYTOR* and EMT/stemness was also validated in HNSCC patients using bulk RNA‐seq data from TCGA database (Figure S3a,b, Supporting Information) and scRNA‐seq data from GEO database (Figure S3c,d, Supporting Information). Notably, AP‐1 is one of the major downstream cascades of MAPK and PI3K‐Akt pathway. Previously, we have demonstrated that FOSL1 is the dominant AP‐1 family member in HNSCC that controls EMT, maintains cancer stemness, and promotes metastasis.^[^ ^9^ ^]^ Thus, based on our bioinformatics analysis, we hypothesized that *CYTOR* may regulate FOSL1 signaling to facilitate the malignant phenotypes of HNSCC. To verify this hypothesis, we performed Western‐blot analysis and revealed that FOSL1 protein levels were significantly downregulated by depletion of *CYTOR* expression (Figure 4d). Conversely, overexpression of *CYTOR* resulted in an upregulation of FOSL1 protein levels (Figure 4d). As expected, RT‐qPCR confirmed that depletion of *CYTOR* significantly suppressed the expression of FOSL1 in SCC1 and HN6 cell lines and overexpression of *CYTOR* promoted FOSL1 expression at transcriptional levels (Figure 4e). Furthermore, luciferase reporter assays showed that AP‐1 activities were inhibited in HNSCC cells with depletion of *CYTOR* (Figure 4f). Collectively, these data indicate that *CYTOR* regulates FOSL1 signaling in HNSCC.

Because *CYTOR* is mainly located in the nucleus of HNSCC cells with high aggressive behavior, we hypothesized that *CYTOR* may form a transcriptional complex with FOSL1 to regulate FOSL1‐dependent transcription. To test this hypothesis, we first examined the physical interaction between *CYTOR* and FOSL1 using RNA pull‐down assay. We utilized two biotinylated‐probes that targeted different regions of *CYTOR* and a biotinylated‐probe of LacZ as the negative control. As shown in Figure 4g, both probes were able to pull down FOSL1, indicating a physical interaction between *CYTOR* and FOSL1. In addition, we performed the reciprocal IP assays using a FOSL1 antibody, followed by RT‐qPCR, and confirmed that *CYTOR* can be co‐immunoprecipitated with FOSL1 antibody (Figure 4h). The co‐localization of *CYTOR* and FOSL1 was also observed in HNSCC cells and tissues (Figure S3e, Supporting Information). These results suggested that *CYTOR* may regulate FOSL1 signaling through physically interacting with FOSL1 to form a transcription complex.

### 2.5 *CYTOR* Associates with FOSL1 to Establish SEs in HNSCC

**Figure 5:**
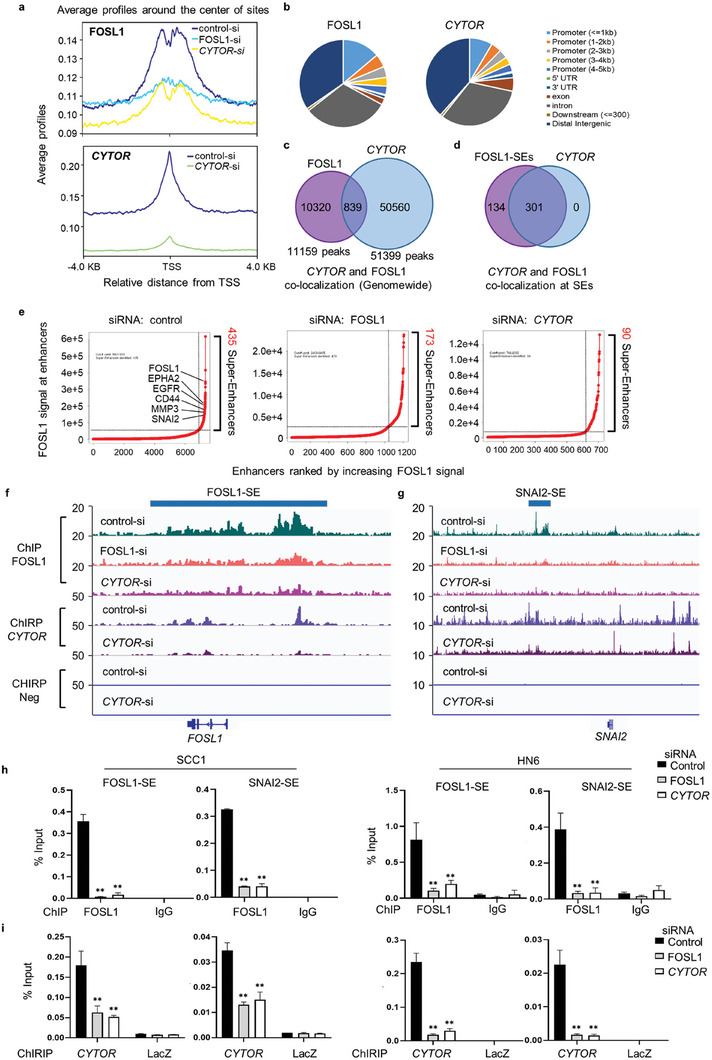
*CYTOR* associates with FOSL1 to establish SEs in HNSCC. a) FOSL1 and *CYTOR* occupancies on genome in SCC1 cells after transfection with control siRNA, FOSL1 siRNA or *CYTOR* siRNA by ChIP‐seq and ChIRP‐seq. b) The percent of different genomic distribution of FOSL1 and *CYTOR* peaks on genome in SCC1 by ChIP‐seq analysis. c) Co‐localization of FOSL1 and *CYTOR* at genome‐wide analyzed by ChIP‐seq and ChIRP‐seq. d) FOSL1‐dependent SEs were identified by ChIP‐seq signal of FOSL1, and *CYTOR* selectively co‐localized with FOSL1 at FOSL1‐dependent SEs. e) Depletion of FOSL1 or *CYTOR* dramatically reduced SEs in SCC1 cells based on ChIP‐seq signaling of FOSL1. f,g) The occupancy of FOSL1 and *CYTOR* at SEs regions of *FOSL1* (F) and *SNAI2* (G) in SCC1 cells treated with control siRNA, FOSL1 siRNA, or *CYTOR* siRNA by ChIP‐seq and ChIRP analysis. h) ChIP‐qPCR showed that depletion of *CYTOR* or FOSL1 by siRNA decreased the recruitment of FOSL1 on the SE regions of *FOSL1* and *SNAI2* in HNSCC cells. **p < 0.01 by one‐way ANOVA. i) ChIRP‐qPCR showed that the recruitment of *CYTOR* on the SE regions of *FOSL1* and *SNAI2* was significantly reduced in HNSCC cells treated with FOSL1 or *CYTOR* siRNA. **p < 0.01 by one‐way ANOVA.

To further investigate the mechanisms of *CYTOR* in FOSL1‐dependent target gene transcription, chromatin immunoprecipitation sequencing (ChIP‐seq) and chromatin association by RNA purification sequencing (ChIRP‐seq) were performed in SCC1 cells. We identified 11159 FOSL1‐occupied regions (peaks) and 51399 *CYTOR* peaks on chromatin in control SCC1 cells. Similar to FOSL1, global analysis of the distribution of *CYTOR*‐occupied regions relative to annotated transcription start sites (TSSs) revealed most of the *CYTOR* peaks were intragenic and intergenic sites located distally from the promoter (**Figure** **5**a,b). This data suggests that, like FOSL1, *CYTOR* predominantly associates with the enhancer regions in HNSCC cells instead of the promoter regions. The analysis showed that 839 co‐localized peaks between *CYTOR* and FOSL1 overlapped (Figure 5c).

Previously, we have shown that FOSL1 is a master regulator that promotes the malignant progression of HNSCC via establishing SEs.^[^ ^9^ ^]^ To investigate the potential roles of *CYTOR* in establishing FOSL1‐dependent SEs (FOSL1‐SEs), we characterized FOSL1‐SEs using the FOSL1‐ChIP signals. As shown in Figure 5d, 435 FOSL1‐SEs were identified in SCC1 cells using ChIP‐seq signal of FOSL1. Among the 435 FOSL1‐SEs, 301 of them contained the peaks of *CYTOR*. This data indicated that *CYTOR* and FOSL1 were majorly co‐localized at FOSL1‐SEs regions. Next, to investigate the critical role of *CYTOR* in establishing SEs, we also examined FOSL1 and *CYTOR* enrichment on chromatin in parallel with *CYTOR* or FOSL1 depleted SCC1 cells. Depletion of *CYTOR* led to a global decrease of FOSL1 recruitment at the chromatin (Figure 5e). More importantly, the majority of FOSL1‐SEs in SCC1 cells were disrupted after knockdown of *CYTOR* (Figure 5e). Of note, only 90 FOSL1‐SEs were identified in *CYTOR* depleted cells, whereas 173 FOSL1‐SEs were identified in FOSL1 depleted cells (Figure 5e).

ChIP‐seq and ChIRP‐seq also identified that FOSL1 and *CYTOR* were enriched in the SE regions of *FOSL1* itself that play a critical role in HNSCC growth, invasion, metastasis and stemness (Figure 5f). *SNAI2* has been demonstrated as a key target gene of FOSL1‐SEs to maintain cancer stemness and promote metastatic properties of HNSCC. The ChIP‐seq and ChIRP‐seq analysis revealed that the signal of *CYTOR* and FOSL1 were highly enriched at the SE region of *SNAI2* that was significantly reduced upon *CYTOR* depletion (Figure 5g). ChIP‐qPCR and ChIRP‐qPCR also confirmed that depletion of *CYTOR* or FOSL1 significantly disrupted the SEs at *SNAI2* and *FOSL1* (Figure 5h,i) in both SCC1 and HN6 cells. In addition, overexpression of *CYTOR* significantly enhanced the recruitment of FOSL1 in the SE regions of *FOSL1*and *SNAI2* (Figure S4a, Supporting Information). Because MED1 and BRD4 are abundant in the region of SEs, we further detect the enrichments of MED1 and BRD4 in the SE regions of *FOSL1* and *SNAI2*. As shown in Figure S4b (Supporting Information), depletion of *CYTOR* significantly dampened the recruitment of MED1 and BRD4 in the SE regions of *FOSL1* and *SNAI2*. As expected, depletion of *CYTOR* did inhibit the mRNA expression of SNAI2 (Figure S4c, Supporting Information). A positive correlation of *CYTOR* and *SNAI2* expression was also observed in HNSCC patients from TCGA dataset (Figure S4d, Supporting Information). Collectively, our data clearly demonstrated that *CYTOR* controls FOSL1‐SEs and its target gene expression in HNSCC.

### 2.6 *CYTOR* Promotes the Formation of FOSL1 Phase‐Separated Condensates

**Figure 6:**
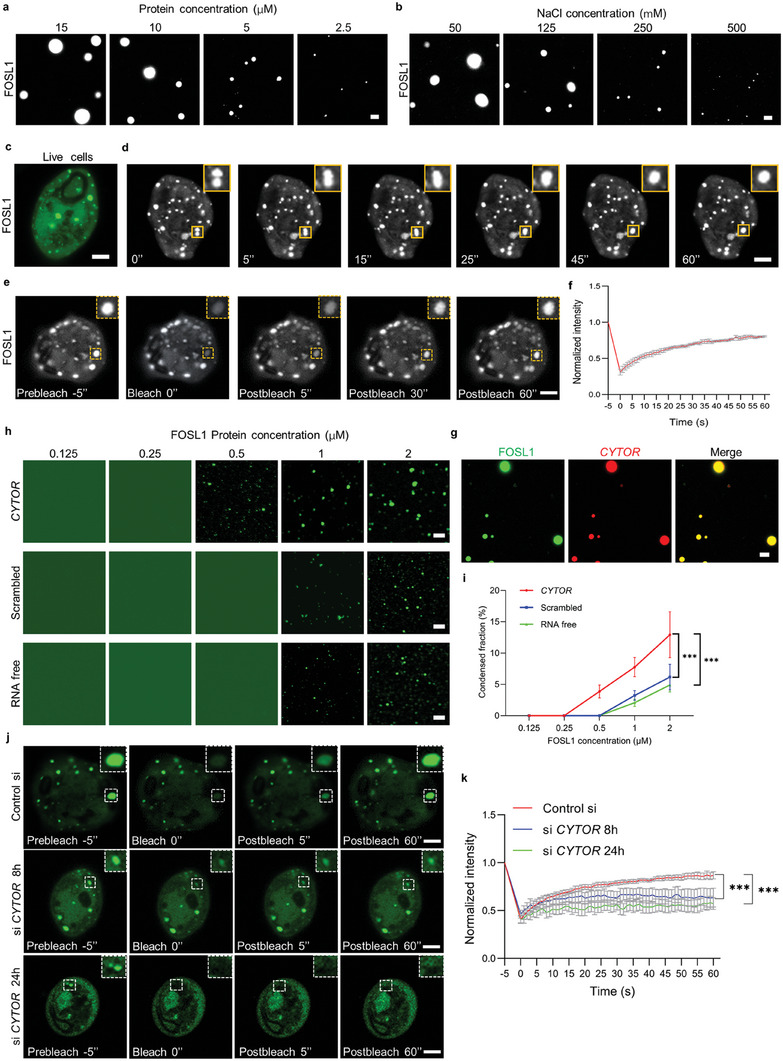
*CYTOR* promotes the formation of FOSL1 phase‐separated condensates. a,b) Representative fluorescence images of phase separated condensates formed by purified FOSL1 protein labeled with Alexa Fluor 488 by in vitro droplet assay, with increasing protein concentration a) and salt concentration b) as indicated. Scale bar, 5 µm. c) Representative fluorescence images of FOSL1 puncta formation in live HNSCC cells transfected with EGFP‐FOSL1 plasmid. Scale bar, 5 µm. d) Time‐lapse fluorescence images showed that FOSL1 droplets underwent fusion event in HNSCC live cells. e) Time‐lapse fluorescence images of FRAP experiment of EGFP‐FOSL1 puncta in live HNSCC cells. Scale bar, 5 µm. f) Quantification of normalized fluorescence intensity of EGFP‐FOSL1 droplets in indicated region of live HNSCC cells with FRAP experiment. g) Fluorescence images showed that mixture of fluorophore‐tagged FOSL1 (10 µm) and Cy3‐labeled *CYTOR* (2 µm) formed phase separated condensates containing both FOSL1 and *CYTOR* in droplet formation assay buffer. Scale bar, 5 µm. h) Fluorescence images of FOSL1 condensates formation at indicated concentration with the presence of *CYTOR* (1 µm), scrambled RNA (1 µm) or the absence of RNA. Scale bar, 5 µm. i) Percentage fluorescence fraction of FOSL1 condensates was measured in droplet formation assay at indicated concentration, mixed with *CYTOR*, scrambled RNA or no RNA. ***p < 0.001 by two‐way ANOVA. j) Time‐lapse fluorescence images of FRAP experiment of EGFP‐FOSL1 puncta in live HNSCC cells transfected with control siRNA and *CYTOR* siRNA at indicated time. Scale bar, 5 µm. k) Quantification of normalized fluorescence intensity of FOSL1 droplets of live HNSCC cells treated with control siRNA or *CYTOR* siRNA at indicated time. ***p < 0.001 by two‐way ANOVA.

Recently, it has been implicated that the formation of phase separation is critical for the SE‐driven gene transcription, which can guarantee the highly concentrated transcriptional machinery at regions of SEs to activate the expression of cell identity genes.^[^ ^10^ ^]^ Interestingly, our FISH and IF experiments revealed that the distribution of *CYTOR* and FOSL1 was not even throughout the nucleus, but instead formed puncta in HNSCC cells, which is a feature of phase‐separated like condensates. This observation suggests that *CYTOR* might interact with FOSL1 to assemble RNA‐protein complexes undergoing liquid‐liquid phase separation (LLPS) at SE regions. To investigate whether FOSL1 puncta are phase‐separated condensates, we first determined whether FOSL1 formed phase‐separated droplets in vitro. As shown in **Figure** **6**a,b, FOSL1 protein labeled with Alexa Fluor 488 was observed to form phase‐separated condensates with a concentration dependent size, ranging from 2.5 to 15 µm in a LLPS buffer solution containing 100 mm NaCl. The size of FOSL1 condensates was found to be smaller as the concentration of NaCl in the LLPS buffer solution was increased, from 50 to 500 mm. Importantly, we observed that FOSL1 did form phase‐separated droplets in live HNSCC cells transfected with FOSL1‐EGFP plasmids (Figure 6c). In addition, proximal droplets were observed to be fused in HNSCC cells, exhibiting characteristic liquid‐like fusion properties of necking and relaxation to spherical shapes (Figure 6d). More importantly, the fluorescence recovery after photobleaching (FRAP) assay showed that FOSL1 puncta were able to recover up to 44.9% fluorescence in 5s, 69.8% in 30s and 80.7% fluorescence in 60s (Figure 6e,f). These findings support the notion that FOSL1 forms LLPS condensates in HNSCC cells.

Subsequently, we further evaluated the interaction between *CYTOR* and FOSL1 in forming phase‐separated condensates. As demonstrated in Figure 6g, the mixture of Cy3‐labeled *CYTOR* and Alexa Fluor 488‐tagged FOSL1 resulted in the formation of LLPS droplets that contained both *CYTOR* and FOSL1 in vitro. Next, we revealed that *CYTOR* promoted the formation of FOSL1 condensates. As shown in Figure 6h,i, obvious droplets were detected at a FOSL protein concentration of 0.5 µM in the presence of *CYTOR*, while no FOSL1 condensates were observed in the absence of *CYTOR*. Additionally, the size of FOSL1 condensates was increased in the presence of *CYTOR* as compared to the scrambled RNA or absence of RNA. These findings indicate that *CYTOR* interacts with FOSL1 and promotes its phase separation in vitro. To reinforce this finding in live cells, we performed the FRAP assay in HNSCC cells with or without knockdown of *CYTOR*. FRAP assays showed that FOSL1 condensates were capable of recovery after photobleaching, but silencing *CYTOR* caused a delay and reduction in their recovery (Figure 6j,k). Taken together, these findings indicate that *CYTOR* plays a crucial role in preserving the phase separated state of FOSL1 condensates in HNSCC cells, which stabilizes FOSL1‐associated SEs and leads to the high expression of FOSL1‐SE driven genes.

### 2.7 CYTOR is Regulated by FOSL1 in HNSCC

**Figure 7:**
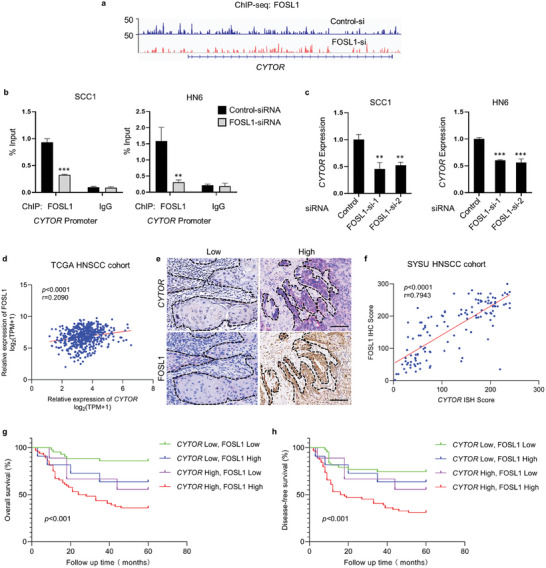
*CYTOR* is regulated by FOSL1 in HNSCC. a) FOSL1 occupancies at the locus of *CYTOR* by FOSL1 ChIP‐seq. b) Knockdown of FOSL1 with siRNA reduced the recruitment of FOSL1 at the promoter of *CYTOR* in HNSCC cells by ChIP‐qPCR. **p < 0.01 and ***p < 0.001 by Student's *t*‐test. c) Depletion of FOSL1 with siRNA suppressed the expression of *CYTOR* in HNSCC cells by RT‐qPCR. **p < 0.01 and ***p < 0.001 by one‐way ANOVA. d) Relation between *CYTOR* and FOSL1 mRNA expression in HNSCC based on TCGA database by Pearson correlation analysis. e) Representative images of FOSL1 IHC staining and *CYTOR* ISH staining in HNSCC tissues. Scale bar, 100 µm. f) Relation between *CYTOR* and FOSL1 protein expression in HNSCC based on ISH and IHC staining by Pearson correlation analysis. g,h) Kaplan‐Meier curves and log‐rank test for overall survival, g) and disease‐free survival, h) of HNSCC patients grouped by the expression of FOSL1 and *CYTOR* from SYSU cohort.

Interestingly, we also observed that FOSL1 was occupied in the promoter of *CYTOR* in our ChIP‐seq data, which was further confirmed by ChIP‐qPCR assay (**Figure** **7**a,b). These results indicate that the expression of *CYTOR* might be regulated by FOSL1 at the transcription level. As expected, RT‐qPCR confirmed that knockdown of FOSL1 did suppress the expression of *CYTOR* (Figure 7c). To reinforce these findings in HNSCC patients, we detected the expression of *CYTOR* and FOSL1 in HNSCC samples. As shown in Figure 7d–f, the *CYTOR* expression was positively correlated with FOSL1 expression in both TCGA dataset and SYSU patient cohort. Importantly, HNSCC patients with high *CYTOR* and FOSL1 expression levels had the poorest overall survival and disease‐free survival (Figure 7g,h). Taken together, we conclude that *CYTOR* and FOSL1 form a positive feedback circuit to promote metastasis and stemness of HNSCC.

### 2.8 Targeting *CYTOR* Suppresses Tumor Growth and Metastasis in HNSCC

**Figure 8:**
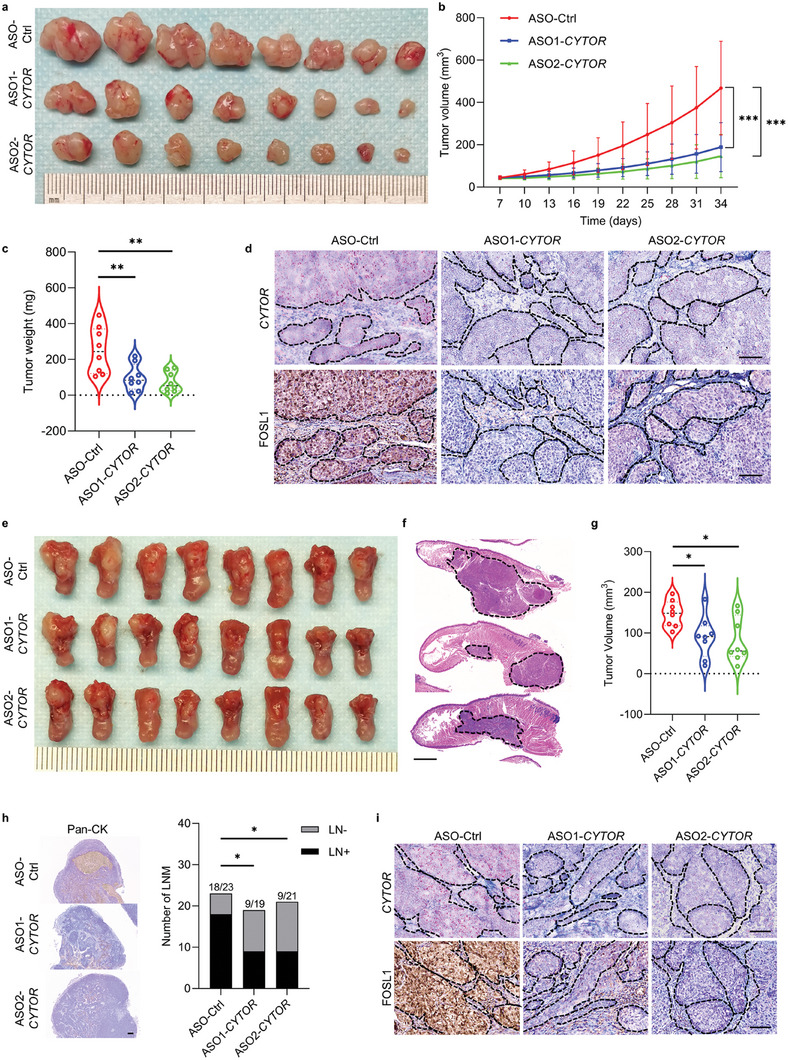
Targeting *CYTOR* suppresses tumor growth and metastasis in HNSCC. a) Targeting *CYTOR* with ASO administration by intra‐tumoral injection suppressed HNSCC subcutaneous xenograft growth. b) Quantification of tumor growth curves for HNSCC subcutaneous xenograft with ASO‐control, ASO1‐*CYTOR*, and ASO2‐*CYTOR* administration. ***p < 0.001 by two‐way ANOVA. c) Quantification of tumor weight of HNSCC subcutaneous xenograft. **p < 0.01 by one‐way ANOVA. d) Representative images of *CYTOR* ISH staining and FOSL1 IHC staining in HNSCC subcutaneous xenograft treated with ASO‐control, ASO1‐*CYTOR*, and ASO2‐*CYTOR*. Scale bar, 100 µm. e) Targeting *CYTOR* with ASO systematically administration by tail vein injection inhibited HNSCC orthotopic xenograft growth. f) Representative images of HE staining of SCC1 formed orthotropic xenograft with ASO‐control, ASO1‐*CYTOR*, and ASO2‐*CYTOR* systematically administration. Scale bar, 1 mm. g) Quantification of tumor volume of HNSCC orthotropic xenograft in ASO‐control, ASO1‐*CYTOR*, and ASO2‐*CYTOR* groups. *p < 0.05 by one‐way ANOVA. h) Representative images of Pan‐CK IHC staining in cervical lymph node from orthotropic tumor bearing mice treated with ASO‐control, ASO1‐*CYTOR*, and ASO2‐*CYTOR*. Scale bar, 300 µm. *p < 0.05 by Chi‐square test. i) Representative images of *CYTOR* ISH and FOSL1 IHC staining in HNSCC orthotropic xenograft with ASO‐control, ASO1‐*CYTOR*, and ASO2‐*CYTOR* systematically administration. Scale bar, 100 µm.

To assess the therapeutic value of targeting *CYTOR* in HNSCC, the HNSCC subcutaneous xenograft models were established and then administered with *CYTOR* antisense oligonucleotide (ASO) by intratumoral injection. As shown in **Figure** **8**a–c, *CYTOR* ASO treatment significantly reduced tumor volume and tumor weight compared with the control ASO in the HNSCC subcutaneous xenograft models. The ISH staining showed that the expression of *CYTOR* was significantly inhibited in the xenografts treated with two different *CYTOR* ASOs, and FOSL1 expression was decreased in those xenografts treated with *CYTOR* ASOs (Figure 8d). To further confirm the above observed tumor response upon *CYTOR* ASO treatment, we assessed the therapeutic efficacy in a HNSCC orthotopic model by administrating *CYTOR* ASO systematically. Consistent with the findings in the subcutaneous xenograft model, a significant decrease in tumor volume was observed in mice treated with both *CYTOR* ASO‐1 and *CYTOR* ASO‐2 as compared to control mice (Figure 8e–g). Importantly, the cervical lymph node metastasis was significantly suppressed in mice upon *CYTOR* ASO treatment (Figure 8h). As expected, reduced expression of *CYTOR* and FOSL1 was also detected in xenografts from the tongue of *CYTOR* ASOs‐treated mice compared with the control mice (Figure 8i).

## 3 Discussion

Increasing studies demonstrate that TB is a reliable prognostic factor in HNSCC and a potential target for cancer treatment. Previously, we first showed that TB was correlated with lymph node metastasis and EMT in HNSCC and then uncovered that the decrease of miR‐320a enhanced aggressiveness of TB cells by targeting SUZ12.^[^ ^2^, ^6^ ^]^ In the current study, we extended our findings to characterize the dynamic transcriptomic signatures of TB in HNSCC during the metastasis cascades. We collected the ANCE, tumor center cells, TB cells, and tumor cells from lymph node metastasis using the laser capture microdissection and then examined their transcriptional profiles. We first revealed that 5 significant dynamic transcript expression patterns were observed in the metastasis cascades of HNSCC (cluster 0, 2, 12, 13, 14). Of note, 479 transcripts were specially decreased in TB cells and enriched in metabolic process, including *CYP3A4, CYP3A5, CYP2C19, CYP2C9, CYP2C18, CYP2C8*, which might be inhibited in the presence of pro‐inflammatory cytokines (such as IL‐6, TNF‐α, TGF‐β, and IFN‐γ).^[^ ^11^ ^]^ Consistent with previous studies,^[^ ^1^, ^3^, ^4^, ^6^ ^]^ we also observed that 460 transcripts in cluster 14 were specially increased in TB cells and enriched in extracellular matrix organization and p‐EMT, including *LAMA3, LAMB3, LUM, COL3A1, TGFB3, MMP2, COL12A1, FN1, MMP12, ITGA5, COL1A2, VCAN*, and *COL1A1*. Importantly, the subset of tumor cells with p‐EMT phenotype and gene signatures were also identified in human and murine primary HNSCC at single cell resolution.^[^ ^8a,b^ ^]^ These findings indicated that TB cells are characterized with p‐EMT and metabolic reprogramming.

To identify the key driver gene that controls the p‐EMT phenotype in TB cells, we focused on cluster 14 and established a Bayesian network algorithm, which suggested that *CYTOR* is a central lncRNA governing TB phenotype. *CYTOR*, also referred to as *LINC00152*, located on chromosome 2p11.2, is a long intergenic non‐coding RNA that is highly expressed in several solid tumors and contributes to cell proliferation, migration and invasion, especially in cancers of the digestive system.^[^ ^12^ ^]^ However, little is known about *CYTOR* in TB cells. We showed that the expression of *CYTOR* was consistent with the expression pattern of cluster 14, which was significantly abundant in TB cells. Interestingly, we noted that *CYTOR* was predominately located at the nucleus in TB cells, suggesting that nuclear expression of *CYTOR* drives aggressiveness of TB cells via an unconventional mechanism. Then, we confirmed that the nuclear expression of *CYTOR* did promote metastasis of HNSCC cells using gain‐of‐function and loss‐of‐function experiments in vitro and in vivo.

Currently, increasing studies revealed that *CYTOR* exerted its function as an oncogene at both the cytoplasm and nuclei by disturbing various signaling pathways.^[^ ^12^, ^13^ ^]^ To dissect the regulatory mechanism of nuclear expression of *CYTOR* in TB cells, RNA‐seq was performed in HNSCC cells with depletion of *CYTOR*, suggesting that FOSL1 has a pivotal role in *CYTOR‐*mediated HNSCC progression. FOSL1 is an AP‐1 transcriptional factor which has been confirmed to promote invasion, stemness and metastasis in HNSCC.^[^ ^8^, ^9^, ^14^ ^]^ Importantly, we showed that FOSL1 enhanced the tumorigenicity and metastasis of HNSCC predominantly via selective association with Mediators to establish SEs in our previous studies.^[^ ^9^, ^15^ ^]^

Interestingly, increasing evidence shows that lncRNAs can form RNA‐protein complex and localize to specific DNA sites to establish a RNA:DNA:DNA triplex, which in turn regulates the associated gene expression.^[^ ^16^ ^]^ More recently, Soibam et al.^[^ ^17^ ^]^ reported a new class of lncRNAs called super‐lncRNAs that target SEs and might function as spatial amplifiers for SE‐associated genes. However, the functional role and regulatory mechanisms of super‐lncRNAs are still unknown in cancer. Surprisingly, we found that *CYTOR* and FOSL1 formed a transcriptional complex and were mainly occupied in the intragenic and intergenic sites located distally from the promoter, implicating that *CYTOR* might cooperate with FOSL1 to establish SEs, which was further confirmed by CHIRP‐seq and ChIP‐seq. As expected, the depletion of *CYTOR* dramatically reduced the FOSL1‐dependent SEs in HNSCC cells. These results demonstrate that *CYTOR* serves as a super‐lncRNA that associates with FOSL1 to establish SEs, which in turn activates cancer stemness and pro‐metastatic genes, such as SNAI2 and FOSL1 itself. Intriguingly, we revealed that *CYTOR* could interact with FOSL1 to form phase‐separated condensates, which contributed to stabilizing SE structures and resulted in transcriptional activation of gene expression. These results suggest that protein–lncRNA phase‐separated condensates have a critical role in regulating key oncogenes transcription and maintaining transcriptional addiction in cancer. Moreover, our results revealed that activation of FOSL1 promoted the transcription of *CYTOR*. Collectively, our discovery indicates that *CYTOR* cooperates with FOSL1 to form phase‐separated puncta and establish a positive regulatory circuit to maintain the activity of FOSL1‐dependent SEs and promote invasion and metastasis of TB cells in HNSCC.

In conclusion, we have discovered that TB cells are characterized with p‐EMT and stemness in HNSCC. *CYTOR* is a master regulator in TB of HNSCC and it exerts function in promoting tumorigenicity and metastasis predominantly via interacting with FOSL1 to form phase‐separated condensates and then activate FOSL1‐dependent SEs. We also demonstrate that *CYTOR* is an attractive strategy to target TB for HNSCC therapy. Collectively, our findings reveal new insights into the key regulatory mechanisms of TB phenotypes and provide a novel therapeutic approach for HNSCC.

## 4 Experimental Section

### Patients and Specimen

Two SYSU patient cohorts diagnosed with HNSCC were used in this study. Nineteen samples from 5 patients were included in cohort 1, and the adjacent non‐cancerous tissues, primary tumor tissues and metastatic lymph nodes were harvested for laser capture microdissection and Affymetrix HTA2.0 array. 127 patients were enrolled in cohort 2 between August 2011 and July 2015 for clinicopathological analysis and validation. All patients with HNSCC received primary tumor resection and neck dissection in Hospital of Stomatology, Guanghua School of Stomatology, Sun Yat‐sun University. None of the included cases underwent radiotherapy or chemotherapy prior to the surgery. Clinicopathological data and follow up data were collected from medical records and telephone interviews, and the TNM stage analysis were subjected to 8th UICC classification system. The study was approved by the ethical committee of the Hospital of Stomatology, Guanghua School of Stomatology, Sun Yat‐sun University.

### Laser Capture Microdissection and Biological Analysis

Ten micrometers thick slides of ANCE, primary tumor tissues and metastatic lymph nodes were prepared and performed with hematoxylin‐eosin staining. And then, images of ANCE, tumor central tissues, tumor budding cells and lymph node metastatic cancer cells were obtained under a microscope, and theses tissues were captured and dissected by laser capture microdissection (ArcturusXT Laser Capture Microdissection Systems, Thermo Fisher). These tissue samples were then pooled for RNA extraction, respectively. Total RNA was extracted by TRIzol Regent (Thermo Fisher, cat. no. 15596018) and purified by RNeasy Micro kit (Qiagen, cat. no. 74004). Extracted RNA was used to reverse transcribed to biotin‐labeled cDNA by Affymetrix WT PLUS Reagent Kit (Affymetrix, cat. no. 902280). Labeled cDNA was hybridized with Affymetrix HTA2.0 array (Affymetrix) and scanned by Affymetrix GeneChip Scanner 3000 (Affymetrix). And the scanned data were subjected to STEM software for dynamic transcript expression patterns analysis. Significant dynamic transcript expression patterns were used for GO and pathway analysis, and Bayesian network algorithm was used to identify the key driver molecules and lncRNA in specific transcriptomic landscape of tumor budding cells.

### Published scRNA‐seq Data Collection and Analysis

The scRNA‐seq data that was analyzed in the study were previously published and are available via the accession codes, including GSE144240,^[^ ^18^ ^]^ GSE103322,^[^ ^8a^ ^]^ and GSE172577.^[^ ^19^ ^]^ These scRNA‐seq datasets were used for evaluating tumor budding signature in distinct HNSCC or SCC cell clusters. Among the above datasets, GSE103322 and GSE144240 have provided the detailed information of malignant cell clusters. For GSE144240 dataset, four malignant cell clusters named basal, differentiating, cycling and tumor‐specific keratinocytes (TSKs) were extracted, and the TB signature scores were evaluated in the four distinct tumor subpopulations by violin plots. And TSKs subpopulation, which were considered as potential drivers of invasion and metastasis at leading edges, displayed the highest TB signature scores. For GSE103322 dataset, p‐EMT scores were evaluated in the ten tumors, which were subsequently classified into p‐EMT low tumors (MEEI6, MEEI20, and MEEI 26) and p‐EMT high tumors (MEEI5, MEEI28, MEEI18, MEEI 22, MEEI25, MEEI17, and MEEI16) based on the p‐EMT scores. TB signature scores were evaluated in the ten tumors by violin plots, which revealed that p‐EMT high tumors showed higher TB signature scores compared with p‐EMT low tumors. For GSE172577 dataset, the specificity information of malignant cells was not clear. First, cells with genes < 200 were removed, and doublets were identified by integration of the results from three methods provided by Puram, Sidharth V et al.^[^ ^20^ ^]^ The remaining cells were used for subsequent analysis by the R package Seurat and Python package large‐scale single‐cell gene expression data analysis (Scanpy). Single cells were classified to malignant and non‐malignant cells by R package CopyKAT^[^ ^21^ ^]^ with default parameters and the CNAs inferred by the Python package InferCNV (https://github.com/icbi‐lab/infercnvpy) with default parameters. And malignant cells identified by the above two methods were extracted and classified into seven clusters. Differentially expressed genes (DEGs) were analyzed at the single‐cell level using the FindAllMarkers function of Seurat. GSEA was performed to explore the subclusters and functional properties of all HNSCC cells clusters by R package clusterProfiler.^[^ ^22^ ^]^ And Cluster 2 (C2‐EMT cluster) showed the significantly up‐regulated hallmark EMT pathways, which was defined as high‐EMT cluster. Finally, TB signature scores were evaluated in the seven HNSCC cells clusters using the AddModuleScore function in Seurat with default parameters, revealing C2‐EMT cluster displaying the highest TB signature.

### RNA In Situ Hybridization (ISH)

The probe (Advanced Cell Diagnostics, #464401) targeting *CYTOR* and RNA‐scope 2.5 HD Reagent Kit‐RED (Advanced Cell Diagnostics, #322360) were purchased from Advanced Cell Diagnostics company, and RNA ISH was performed according to the manufacturer's instructions to detect *CYTOR* expression in tissue section. In brief, sections were deparaffinized and incubated with hydrogen peroxide. And then, sections were maintained in retrieval reagents solution and incubated with protease solution. Sections were hybridized with probe at 40 °C for 2 h, and were incubated with amp1, amp2, amp3, amp4, amp5, and amp6. Finally, sections were stained with Fast‐RED solution for 10 min at room temperature and counterstained with hematoxylin. The signals were scored based on the manufacturer's instructions. The images of sections were acquired with the Aperio AT2 Scanner (Leica Biosystems, Germany), and the data were analyzed by Imagescope software.

### Cell Lines and Cell Culture

The human HNSCC cell lines CAL33, SCC15, SCC1, and HN6 were used in the study. CAL33 and SCC15 were purchased from American Type Culture Collection (ATCC, Rockville, MD, USA). SCC1 and HN6 were obtained from the University of Michigan and Wayne State University respectively. CAL33 cells were maintained in DMEM medium (Thermo Fisher, C11995500BT). SCC1, HN6, and SCC15 were maintained in DMEM/F‐12 medium (Thermo Fisher, C11330500BT). The DMEM and DMEM/F‐12 medium were supplemented with 10% fetal bovine serum (FBS, Thermo Fisher, 10099‐141C) and 1% penicillin–streptomycin (Thermo Fisher, 15140122), and all cells were maintained in an incubator at 37 °C in a humidified atmosphere with 5% CO_2_.

### RNA Fluorescence In Situ Hybridization (FISH) and Immunofluorescence

RNA FISH was performed according to RNA‐scope Multiplex Fluorescent Reagent Kit (Advanced Cell Diagnostics, 323110) manufacturer's instructions. Briefly, HNSCC cells were seeded in culture slides (Life Science Research, Millicell, PEZGS0496) overnight, and cells were fixed with paraformaldehyde solution and incubated with 50%, 70%, and 100% ethanol. After washed with PBS solution and incubated with hydrogen peroxide and protease solution, the cells were hybridized with probe (Advanced Cell Diagnostics, 464401) at 40 °C for 2 h. And then, cells were incubated with amp1, amp2, amp3, and HRP‐C1, and washed with wash buffer before stained with Opal 570 (Asbio Technology Inc., ASOP570) and DAPI. For immunofluorescence, cells were incubated with primary antibody overnight, and then incubated with secondary antibody (Cell Signaling, 4412S) for 1 h before stained with DAPI. Primary antibodies used in immunofluorescence were anti‐Actin (Abcam, ab179467), anti‐FOSL1 (Abcam, ab232745). FISH and immunofluorescence images were obtained by the confocal microscopy imaging system (LSM780, Zeiss).

### Cytoplasmic and Nuclear Fraction, RT‐qPCR and RNA Sequencing

Cytoplasmic and nuclear fraction assay was performed with SurePrep Nuclear or Cytoplasmic RNA Purification Kit (Fisher, BP2805) according to the manufacturer's instructions. In brief, pre‐chilled lysis buffer was added into cells, incubated on ice and transferred to a tube. After centrifugation, the supernatant was transferred to a new pre‐chilled tube for cytoplasmic RNA extraction and the pellet was used for nuclear RNA extraction. For RT‐qPCR assay, total RNA was extracted with TRIzol Regent (Thermo Fisher, 15596018), purified by RNeasy Micro kit (Qiagen, 74004), and transcribed into cDNA by PrimeScript RT reagent Kit (Takara, RR047A) according to manufacturer's instructions. RNA was quantified by SYBR Green PCR Master Mix (Yeasen, 11201ES08) on a LightCycle 96 System (Roche). All primers were designed referring to GeneBank data, and the sequences were presented in Table S1 (Supporting Information). For RNA‐seq, MGISEQ2000 platform (MGI Technology, MGISEQ‐2000) were employed for library preparation and sequencing. The RNA‐seq reads were aligned to the GRCh37 (hg19) human reference genome for mapping with Bowtie. The gene expression level was evaluated and differentially expressed genes were determined with edgeR package. Genes that showed altered expression with > 2‐fold change and *p* < 0.05 were considered as differential expression and were performed with GO analysis. GSEA software (https://www.broadinstitute.org/GSEA) were employed to rank the significant differentially expressed genes and to analyzed statistical data.

### Lentiviral Plasmid and Silence RNA (siRNA) Transfection

For lentiviral plasmid transfection, the shRNA targeting and overexpressed *CYTOR* sequences were presented in Table S1 (Supporting Information). The lentivectors were mixed with packaging plasmids (vsvg and Δ8.9) and Lipofectamine 2000 (Thermo Fisher, 11668019), and then co‐transfected into 293T cells. After 48 h, the supernatant of 293T cells medium was harvested and used as lentivirus to establish *CYTOR* knock‐down or overexpressed stable HNSCC cell lines. SCC1, HN6, CAL33, and SCC15 were incubated with lentivirus solution for 24 h. And puromycin (2 mg L^−1^) was used to select stable cell lines for 2 days after 48 h lentivirus infection. For transient siRNA transfection, the siRNA oligonucleotides sequences were presented in Table S1 (Supporting Information). And HNSCC cells were transfected with siRNA by Lipofectamine RNAiMAX (Thermo Fisher, 13778150) according to the manufacturer's protocol.

### Sphere Formation Assay and Limiting Dilution Assay

Cells were seeded in ultra‐low attachment 96‐well plates at the indicated cell density (1000, 500, 200, 100, and 50) in serum‐free DMEM/F‐12 with 2% B27 supplement (Thermo Fisher, 17504044), human recombinant EGF (20 ng ml^−1^; PrimeGene, 105‐04) and human recombinant basic FGF (10 ng ml^−1^; PrimeGene, 104‐02) in a 37 °C humidified incubator with 5% CO_2_. For each cell density, at least 5 wells were seeded with cells. Ten days later, the number of spheres was counted in each well under a microscope, and the diameter of sphere was measured with ImageJ software, and the frequency of cancer stem cell was computed by ELDA software (http://bioinf.wehi.edu.au/software/elda/).

### Wound Healing Assay and Invasion Assay

For the wound healing assay, HNSCC cells were seeded into a 6‐well plate and maintained in the incubator. Wounds were scratched in the cell monolayers with a pipette tip when the cell confluence reached 90%. And the cells were imaged under a microscope at the indicated time (0, 12, 16, and 24 h). Distance of cell migration and data analysis were performed by imageJ software. Cell invasion were evaluated by trans‐well assay. The cells were seeded in the upper chambers (Corning) coated with Matrigel (Corning, 354234) and cultured in 200 µl serum‐free DMEM/F‐12 medium, and 10% FBS was added in the lower chambers. After 24 h of incubation, the cells were stained with crystal violet (Sigma, C3886), and the number of cells that invaded to the lower side of the inserts was counted under a microscope.

### Animal Studies

All animal experiments were approved by Institutional Animal Care and Use Committee (IACUC), Sun Yat‐Sen University (Approval no. SYSU‐IACUC‐2021‐000585). Four to five‐week‐old female BALB/c nude mice were obtained from Animal Laboratory Center, Sun Yat‐sen University. For HNSCC orthotopic xenograft model establishment, 1.5 × 10^6^ SCC1 cells (Control sh, *CYTOR* sh1, and *CYTOR* sh2) and CAL33 cells (Lenti‐*CYTOR* and Lenti‐Ctrl) were mixed with DMEM/F‐12 and Matrigel mixtures and injected into the tongue of nude mice. The xenografts were harvested for tumor volume (V) calculation with the following formula: V = Length × width^2^/2, and lymph nodes were dissected and collected for local metastasis evaluation after the mice were sacrificed. For topical intra‐tumoral therapeutic models, 1.5 × 10^6^ SCC1 cells were mixed with DMEM/F‐12 and Matrigel mixtures and implanted into the right axilla of nude mice to generate HNSCC subcutaneous xenografts, and the mice were randomly divided into 3 groups (ASO‐Ctrl, ASO1‐*CYTOR*, and ASO2‐*CYTOR*) for indicated intra‐tumoral ASO administration after palpable subcutaneous xenografts formation. For intra‐tumoral ASO treatment, 5 nmol ASO reagent (Ribobio, U0809) dissolved with PBS solution was administrated with intra‐tumoral injection every 3 days for 4 weeks. The tumor volume (V) was calculated every 3 days with the recorded tumor length and width by following the formula: V = Length × width^2^/2, and tumor growth curve was plotted based on tumor volume. And anti‐tumor effect of intra‐tumoral treatment was evaluated by the tumor growth curve and tumor weight. For systematic therapeutic models, 1.5 × 10^6^ SCC1 cells were mixed with DMEM/F‐12 and Matrigel mixtures and injected into the tongue of nude mice to establish HNSCC orthotopic xenografts, and the mice were randomly divided into 3 groups (ASO‐Ctrl, ASO1‐*CYTOR*, and ASO2‐*CYTOR*) for indicated systematic ASO administration after 7 days. For systematic ASO treatment, 10 nmol ASO reagent (Ribobio, U0809) dissolved with PBS solution was administrated with tail intravenous injection every 3 days for 3 weeks. After the mice were sacrificed, xenografts and lymph nodes were harvested for tumor volume and local lymph node metastasis evaluation respectively. Collected xenografts were stained with HE for tumor formation validation and tumor budding assessment, and ISH and IHC assays were performed for expression of *CYTOR* and FOSL1 evaluation. And Pan‐CK IHC assays were performed on lymph node to evaluate local metastasis.

### TCGA Data Collection and Analyses

UCSC Xena browser (https://xenabrowser.net) was used to down‐load RNA sequence and clinicopathological data of TCGA HNSCC cohort. First, Data related to *CYTOR* and FOSL1 mRNA expression were extracted and integrated with R package, and associated clinical and survival data were used for validating clinicopathological significance of *CYTOR* in HNSCC. Pearson correlation analysis was performed to evaluate the relation between *CYTOR* and FOSL1 mRNA expression in TCGA HNSCC cohort. Second, genes involved in cluster14 were defined as TB signature genes, and the samples were scored based on the expression of genes included in cluster14. The samples were classified into TB signature scores high and TB signature scores low by TB signature genes expression using a median‐based threshold approach. Kaplan‐Meier curves and log‐rank test were performed for evaluating overall survival of HNSCC patients grouped by TB signature genes expression. Subsequently, genes expressed level in TB signature high and TB signature low groups were evaluated. Genes with the value of log_2_(fold change) > 1 and the adjusted *p*‐value < 0.05 were identified as DEGs. And DEGs were subjected to subsequent GO and KEGG pathways analysis.

### Immunohistochemistry (IHC)

IHC assay was performed with Anti‐Rabbit and Anti‐Mouse HRP/DAB (ABC) IHC DETECTION Kit (Abcam, ab64261 and ab64259) according to the manufacturer's instructions. The primary antibodies used in IHC were anti‐Pan‐Keratin (Cell Signaling, 4545S), anti‐FOSL1 (Abcam, ab232745). Images of specimen sections were obtained with the Aperio AT2 Scanner (Leica Biosystems, Germany), and the data were analyzed by imageJ software. The IHC score was calculated by the formula as the addition of product of different density and percentage of cell staining.

### Western Blotting

The total protein was extracted by RIPA lysis buffer (CWBIO, CW2333S) mixed with cocktail inhibitors on ice, and protein concentration was quantified with BCA assay kit (CWBIO, CW0014S) according to manufacturer's instructions. Protein was separated by SDS‐PAGE gel and then transferred to PVDF membranes (Millipore, ISEQ00010), and then the membranes were incubated with 5% skimmed milk in TBST and hybridized with primary antibodies overnight at 4 °C. After incubated with secondary antibodies at room temperature, and protein was detected using chemiluminescence detection reagent (Millipore, WBKLS0500), and the bands were analyzed with imaging system (Syngene). The primary antibodies were anti‐FOSL1 (Abcam, ab232745) and anti‐Tubulin (Abcam, ab7291).

### Luciferase Reporter Assay

HNSCC stable AP1 luciferase reporter cell lines were established by lentivirus transfection, and were selected with puromycin (2 mg ml^−1^) for 4 days. After 48 h transfection with indicated siRNAs, luciferase assays were performed with Dual‐Luciferase Assay System (Promega) according to the manufacturer's instructions, and reporter translation activity was evaluated by Renilla luciferase activity normalized with firefly luciferase.

### ChIP‐qPCR and SE Analysis

For ChIP assays, 10^6^ HNSCC cells were used for each ChIP reaction mixture. Cells were cross‐linked with 1% formaldehyde solution for 10 min at 37 °C, neutralized by glycine, and lysed with lysis buffer. Total cell lysate was sonicated to 200 to 400 bp DNA fragments. Sonicated chromatin was incubated with indicated antibodies overnight at 4 °C. And the DNA was separated and purified from immunoprecipitated complexes, and was measured by qPCR. Data were presented as the percentage of input DNA, and the primer sequences used in ChIP‐qPCR were listed. SEs analysis was performed with ROSE software (https://bitbucket.org/young_computation/rose). Briefly, the FOSL1 peaks within 12.5 kb were sewed together as the enhancer clusters. Enhancer clusters were ranked and plotted according to each FOSL1 ChIP‐seq signal, and sewed enhancer clusters that surpassed infection point were identified as SEs. Peaks that were included within ±2 kb from a RefSeq TSS were eliminated. Enhancers were assigned to RefSeq transcript with TSS that was closest to the center of the enhancer. The intersect utility in bedtools was performed to evaluate the overlapping of the SEs with other SEs or enhancers. The minimum overlap requirement is 1 bp. And the TE‐ and SE‐associated genes were determined with the ROSE framework. The fold change of SE‐associated genes and TE‐associated genes was compared with a *t*‐test.

### ChIRP and ChIRP‐qPCR

Biotin tagged *CYTOR* antisense DNA probes were designed and synthesized by LGC Biosearch Technologies. Cells were cross‐linked with 1% formaldehyde solution for 10 min at 37 °C, neutralized by glycine, and lysed with lysis buffer. The lysate was sonicated to 200 to 400 bp DNA fragments. Sonicated chromatin fragments were hybridized with *CYTOR* biotin labeled probes overnight for 4 h at 37 °C. The DNA was separated and purified from immunoprecipitated complexes, and was measured by qPCR (ChIRP‐qPCR).

### ChIP‐seq and ChIRP‐seq

ChIP‐seq and ChIRP‐seq assays were performed with the NEBNext Ultra II DNA Library Prep Kit (NEB, E7103S) following the manufacturer's protocol. In brief, ChIP and ChIRP DNA purification, end repair, A‐tailing, adaptor ligation and PCR amplification were performed with manufacturer's protocol. Multiplexing samples were performed with NEB adaptors (NEB, NEBNext Multiplex Oligos for Illumina, E7335S). Illumina HiSeq 3000 was used for sequencing the library samples, and Illumina Bcl2fastq2 v 2.17 program was used for demultiplexing. To remove adaptors and trim quality bases, Trimmomatic was performed, and low‐quality bases was also eliminated to meet the requirements for quality assessment and ENCODE standards. ChIP‐seq and ChIRP‐seq reads were aligned to the GRCh37 (hg19) human reference genome using Bowtie. Unique mapped reads were subjected to MACS2 for downstream peak calling analysis with default parameters (*p* < 10^−4^). Bigwig files were generated with the bamCoverge utility in deepTools. Peak annotation was generated with R package ChIPseeker, and cis‐regulatory elements annotation system (CEAS) was used to assign ChIP‐seq and ChIRP‐seq enriched regions (peaks) to genes via creating average profiling of all RefSeq genes and overlaps of significant peaks with genomic annotation regions. Genes with significant peaks within 5 kb of their TSSs were regarded as bound. And HOMER was employed to find the most enriched motifs in a given peak set.

### RNA Pull‐Down and Western Blotting

Biotin labeled *CYTOR* was synthesized in vitro by PCR using T7 RNA polymerase with Ribo RNAmax‐T7 Biotin Labelling Transcription Kit (Ribobio, C11002‐1) following the manufacturer's protocol. And the purified biotin labeled *CYTOR* was incubated with SCC1 and HN6 cells lysis, and was pulled down by streptavidin magnetic beads for 1 h. The precipitated proteins were washed, eluted and boiled in SDS sample buffer. The enriched protein was identified by silver staining with mass spectrometry, and was performed for subsequent Western blotting.

### RIP‐qPCR

RIP was performed using EZ‐Magna RIP RNA‐Binding Protein Immunoprecipitation Kit (Millipore) with the manufacturer's instructions. In brief, cells were lysed in RIP lysis buffer on ice and transferred at −80 °C to aid lysis. The lysate was thawed and incubated with Dynabeads protein A/G (Millipore), and then incubated with indicated antibodies or Rabbit IgG at 4 °C overnight. RNA‐protein complex was pulled down by Dynabeads protein A/G, and RNA was separated from beads‐RNA‐protein complex and was extracted for subsequent qPCR assay.

### Expression, Purification and Labeling of FOSL1 Proteins

His tagged FOSL1 constructs were transfected in *Escherichia coli* BL21 (DE3) cells and cultured at 37 °C to an optical density of 0.6. The expression of proteins was induced by 0.2 mm IPTG under 16 °C for 18 h, and cells were collected with centrifugation. After removing supernatant, cells were resuspended and lysed in buffer containing 200 mm NaCl, 50 mm Tris‐HCl PH 7.4, 20 mm imidazole, DNase I and complete protease inhibitor. After sonication, the lysate was centrifugated at 12,000 ***g*** for 30 min to remove debris, and the supernatant was added to 5 ml HisTrap HP column (GE Healthcare) pre‐equilibrated with lysis buffer. And then the column was extensively washed with lysis buffer containing 300 mm imidazole to elute proteins. The combination of fraction containing proteins was concentrated and added to superdex 200pg column (GE Healthcare) with wash buffer. The proteins were collected based on the SDS‐PAGE, and the concentration of protein was measured by Nanodrop (Thermo Fisher), and the purified proteins were maintained at −80 °C for subsequent experiments. FOSL1 protein was labeled with Alexa Fluor 488 by Alexa Fluor 488 Conjugation Kit (Abcam, ab236553) according to manufacturer's protocol.

### In Vitro Droplet Assay

Alexa Fluor 488 tagged FOSL1 protein solution or Cy3 labeled *CYTOR* was diluted at varying concentrations with indicated final NaCl concentration. And the protein or RNA solution was quickly mixed in phase separation buffer containing 10% glycerol, 50 mm Tris‐HCl PH 7.4 and 1mm DTT with crowding agents 10% PEG‐8000, and incubated for 5 min. Then the mixture was pipetted on a chamber formed by a slide with a coverslip fixed with double‐sided tape strips. And slides were imaged with a confocal microscope using a 63× objective (LSM780, Zeiss).

### Fluorescence Recovery after Photobleaching (FRAP) and Live Cell Imaging

FRAP experiments were performed on the confocal microscopy imaging system (LSM780, Zeiss). SCC1 cells were transfected with EGFP‐FOSL1 and imaged on a confocal microscope using 63× oil immersion objective. Photobleaching of phase separated droplets were performed with a 488 nm laser line using 98% laser power. Time‐lapse images for monitoring fluorescence recovery were collected with 1 s interval for 100 s after bleaching. The fluorescence intensity of the phase separated droplets was measured and analyzed by ZEN software, and recovery curves were plotted with FRAP data using Prism 9.0 (GraphPad Software).

### Statistical Analysis

Statistical analysis was performed with Prism 9.0 (GraphPad Software). Data were presented as the mean ± SD. Two‐tailed Student's *t*test and analysis of variance (ANOVA) were performed for comparing mean values, and the difference was considered statistically significant with *p < 0.05, **p < 0.01, and ***p < 0.001. Kaplan‐Meier method was used to plot survival curves and the difference was compared by log‐rank test. Fisher's exact test and Chi‐square test were performed to evaluate the difference between categorical variables. Pearson correlation analysis was performed to evaluate the relation between two variables.

### Ethics Approval and Consent to Participate

This study was approved by the ethical committee of the Hospital of Stomatology, Guanghua School of Stomatology, Sun Yat‐Sun University (Approval no. KQEC‐2020‐13), and informed written consent of all participants were obtained.

## Conflict of Interest

The authors declare no conflict of interest.

## Author Contributions

W.W., B.Y., and R.G.H. contributed equally to this work. C.W., J.L., and W.W. conceived the study. W.W., B.Y., R.G., and G.X. performed most experiments and data analyses. W.W., B.Y., Z.M., C.Y., and M.Z. contributed to the experiments, data acquisition, and/or analysis. N.X. performed the histopathological review. C.W., J.L., and X.L. supervised the study. C.W., J.L., and W.W. wrote the manuscript, which the other authors edited and approved.

## Supporting information

Supporting Information

## Data Availability

The data that support the findings of this study are available from the corresponding author upon reasonable request.
