# Supplementary material for: CYTOR Facilitates Formation of FOSL1 Phase Separation and Super Enhancers to Drive Metastasis of Tumor Budding Cells in Head and Neck Squamous Cell Carcinoma
Source: Adv Sci (Weinh). 2023 Nov 30;11(4):2305002. doi: 10.1002/advs.202305002 (PMC10811474; doi:10.1002/advs.202305002)
Supplement: Supplementary file 1 — Supporting Information [file ADVS-11-2305002-s001.pdf]

ADVANCED SCIENCE
Open Access

Supporting Information

for *Adv. Sci.*, DOI 10.1002/advs.202305002

CYTOR Facilitates Formation of FOSL1 Phase Separation and Super Enhancers to Drive Metastasis of Tumor Budding Cells in Head and Neck Squamous Cell Carcinoma

Wenjin Wang, Bokai Yun, Rosalie G Hoyle, Zhikun Ma, Shadid Uz Zaman, Gan Xiong, Chen Yi, Nan Xie, Ming Zhang, Xiqiang Liu, Dipankar Bandyopadhyay, Jiong Li and Cheng Wang**

Supporting Information

CYTOR Facilitates Formation of FOSL1 Phase Separation and Super Enhancers to Drive Metastasis of Tumor Budding Cells in Head and Neck Squamous Cell Carcinoma

Wenjin Wang1,2,3#, Bokai Yun1,2,3#, Rosalie G Hoyle4#, Zhikun Ma4, Shadid Uz Zaman4, Gan Xiong1,2,3, Chen Yi1,2,3, Nan Xie1,2,3, Ming Zhang1,2,3, Xiqiang Liu5, Dipankar Bandyopadhyay6,7, Jiong Li4,7,8,9, Cheng Wang1,2,3**

The Supporting Information includes 4 Supplementary Figures and one Supplementary Table.

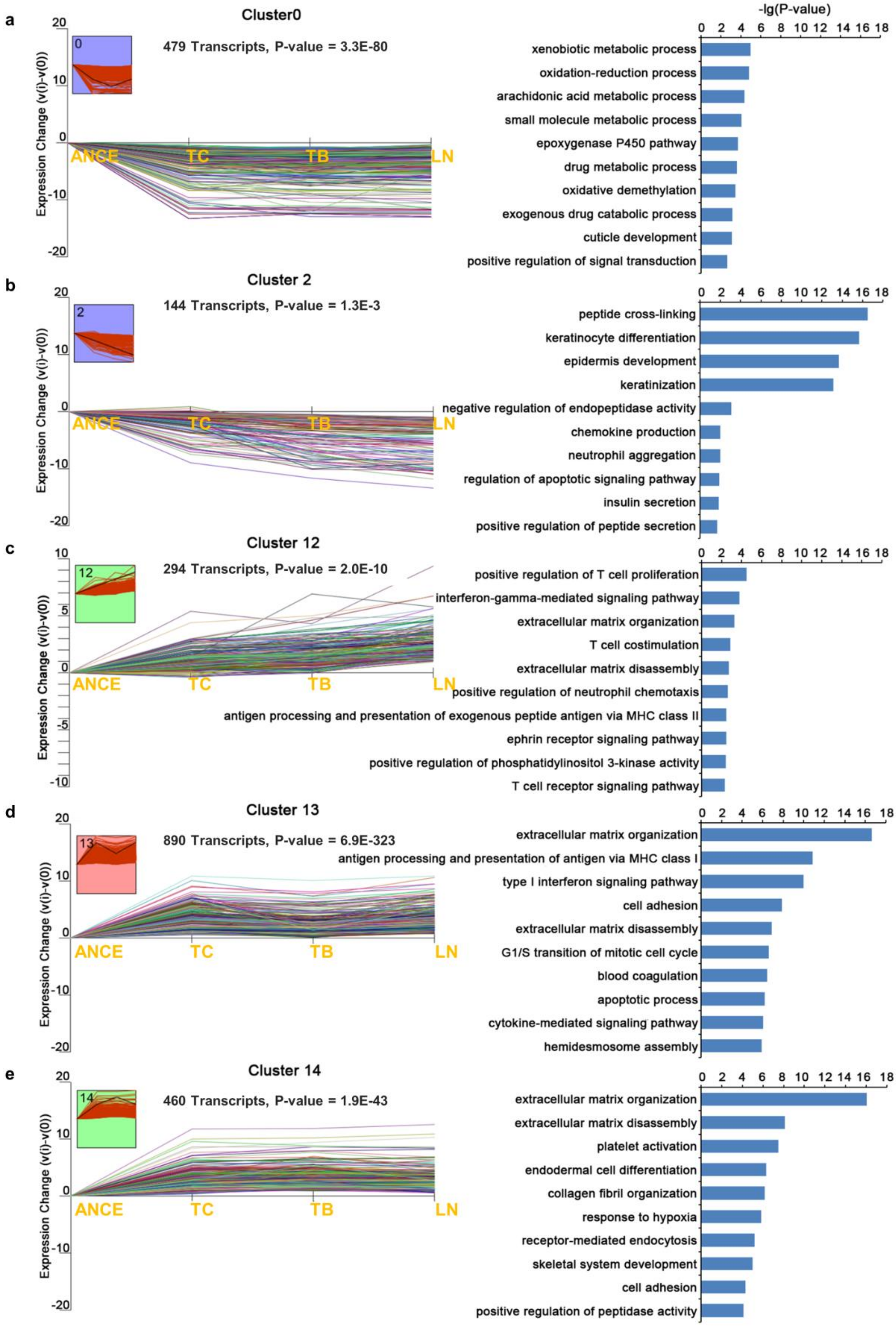

Figure S1, related to Figure 1. GO analysis for significant dynamic transcript expression patterns during the progressive cascade of HNSCC. (**a**) GO analysis

for cluster 0 displaying a decreased trend with a bottom point at tumor budding by STEM analysis. (**b**) GO analysis for cluster 2 with a consistent decreased trend by STEM analysis. (**c**) GO analysis for cluster 12 with a consistent increased trend during the progressive cascade of HNSCC by STEM analysis. (**d**) GO analysis for cluster 13 showing an increased trend with a bottom point at tumor budding by STEM analysis. (**e**) GO analysis for cluster 14 displaying an increased trend with a peak point at tumor budding by STEM analysis.

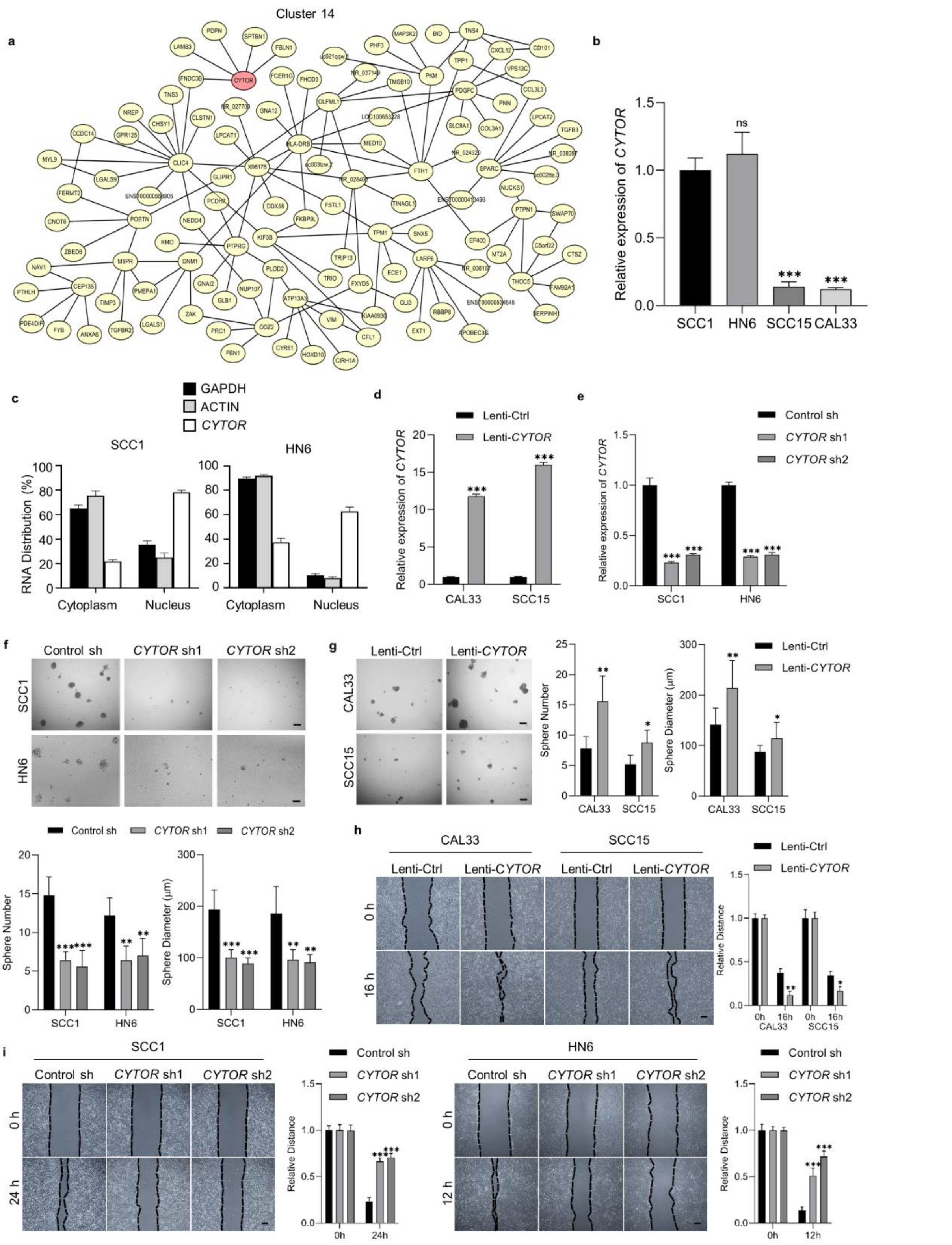

Figure S2, related to Figure 2. *CYTOR* is mainly enriched in nucleus and promotes HNSCC cells stemness and migration. (**a**) Identification of *CYTOR* as the hottest hub lncRNA among genes in cluster 14 through a novel bayesian network

algorithm. (**b**) Quantification of *CYTOR* expression in HNSCC cell lines SCC1, HN6, SCC15 and CAL33. ***p < 0.001 by one-way ANOVA. (**c**) Quantification of *CYTOR* subcellular distribution in HNSCC cell lines SCC1 and HN6 by cytoplasmic and nuclear fractionation and RT-qPCR. (**d**) Quantification of expression of *CYTOR* in HNSCC cells transfected with *CYTOR* overexpression plasmid or control plasmid. ***p < 0.001 by Student's t test. (**e**) Quantification of expression of *CYTOR* in HNSCC cells with/without depletion of *CYTOR* by shRNA. ***p < 0.001 by one-way ANOVA. (**f**) Representative images and quantification of number and diameter of spheres derived from SCC1 and HN6 with/without depletion of *CYTOR* by shRNA. Scale bar, 200μm. **p < 0.01 and ***p < 0.001 by one-way ANOVA. (**g**) Representative images and quantification of number and diameter of spheres derived from CAL33 and SCC15 with/without overexpression of *CYTOR*. Scale bar, 200μm. *p < 0.05 and **p < 0.01 by Student's t test. (**h**) Representative images and evaluation of migration in HNSCC cells with overexpression of *CYTOR* by wound healing assay. Scale bar, 200μm. *p < 0.05 and **p < 0.01 by Student's t test. (**i**) Representative images and evaluation of migration in SCC1 andHN6 with depletion of *CYTOR* by wound healing assay. Scale bar, 200μm. ***p < 0.001 by one-way ANOVA.

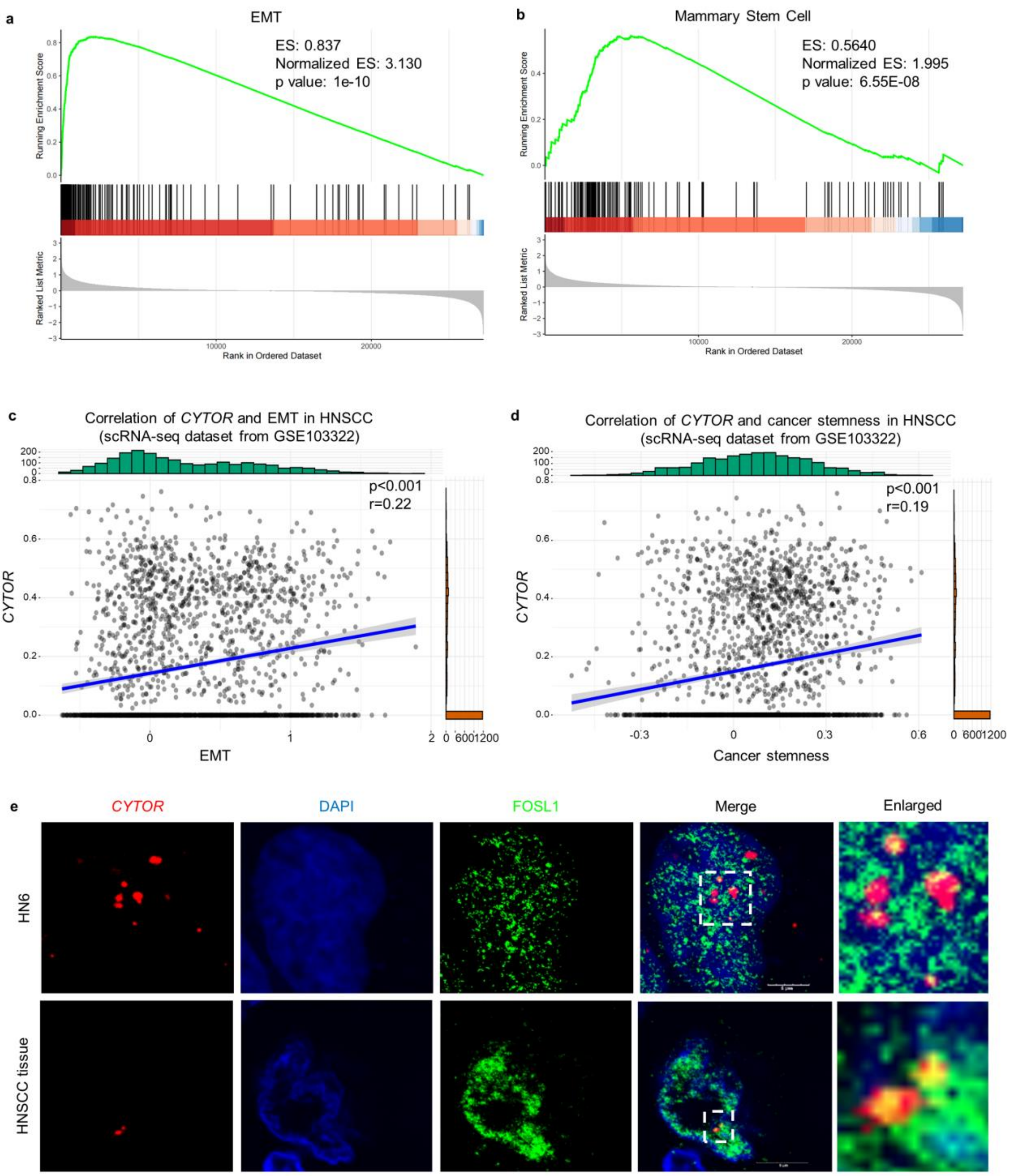

Figure S3, related to Figure 4. *CYTOR* co-localized with FOSL1 and correlates with pEMT and cancer stemness in HNSCC. (**a**) A positive correlation between the abundance of *CYTOR* and EMT was revealed by GSEA in HNSCC tissues from TCGA cohort. (**b**) GSEA revealing the abundance of *CYTOR* correlating with mammary stem cells in HNSCC based on TCGA database. (**c,d**) A positive correlation between the expression of *CYTOR* and EMT (**c**) /cancer stemness (**d**) was revealed based on

scRNA-seq data from GSE103322 dataset by Pearson correlation analysis. (**e**) Detection of expression and subcellular localization of *CYTOR* and FOSL1 in HNSCC cells and primary tumor tissues by FISH and IF experiments, *CYTOR* (red) and FOSL1 (green). Scale bar, 5μm.

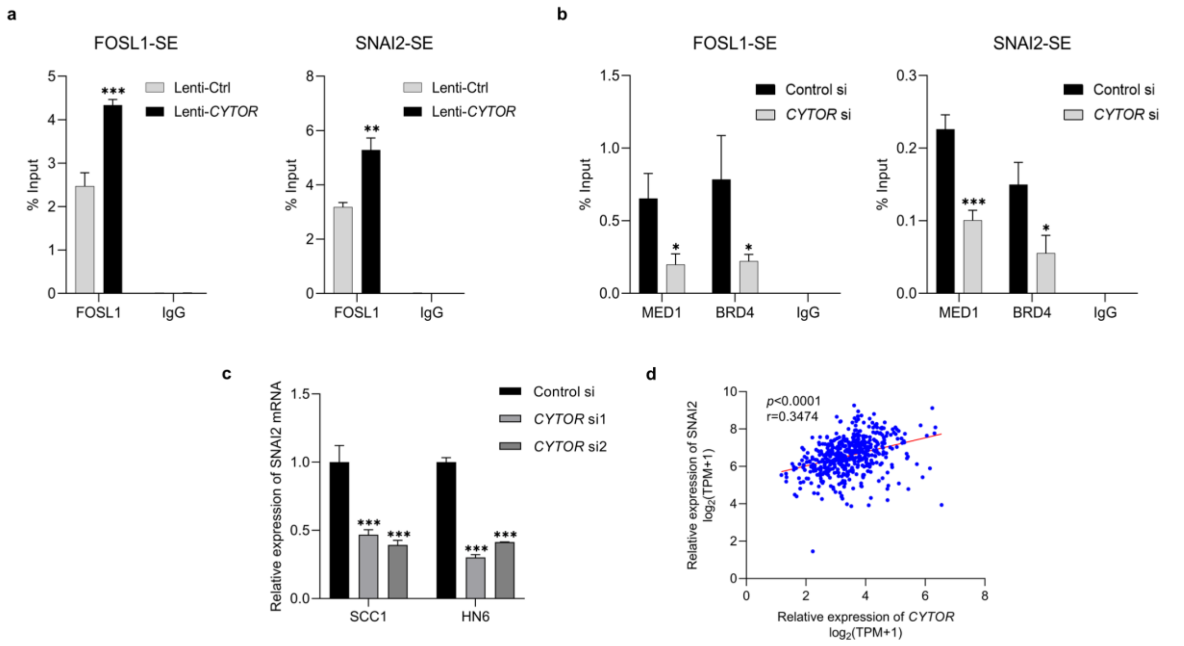

Figure S4, related to Figure 5. ***CYTOR*** **recruits FOSL1 and co-activators MED1 and BRD4 at the region of SEs.** (**a**) Overexpression of *CYTOR* enhanced the recruitment of FOSL1 on FOSL1-SE and SNAI2-SE. **p < 0.01 and ***p < 0.001 by Student's t test. (**b**) Depletion of *CYTOR* significantly reduced the recruitment of BRD4 and MED1 on FOSL1-SE and SNAI2-SE. *p < 0.05 and ***p < 0.001 by Student's t test. (**c**) RT-qPCR showed that SNAI2 transcription were suppressed by depletion of *CYTOR* in SCC1 and HN6, ***p < 0.001 by one-way ANOVA. (**d**) Relation between *CYTOR* and SNAI2 mRNA expression in HNSCC based on TCGA database by Pearson correlation analysis.

Supplementary Table 1. Oligonucleotides sequences

Gene	Application	Oligonucleotides sequences	Source
CYTOR	qPCR	Forward 5′-AGCCCCTGGAAACCTCTTGACTCT-3′<br>Reverse 5′-GAGGCTGGCAAGTTTCCAATATAC-3′	Generay
FOSL1	qPCR	Forward 5′-GCCTGTGCTTGAACCTGA-3′<br>Reverse 5′-TGCTGCTACTCTTGCGATG-3′	Generay
SNAI2	qPCR	Forward 5′-CGAACTGGACACACATACAGTG-3′<br>Reverse 5′-CTGAGGATCTCTGGTTGTGGT-3′	Generay
CYTOR	RIP-qPCR	Forward 5′-AATGCAGCTGAAAGATTCCCTGG-3′<br>Reverse 5′-AAATATCACAGGCAGACCACCCG-3′	Sigma
FOSL1-SE	ChIP-qPCR	Forward 5′-GTCTCCGTGCCCGCTTTCTCTCCT-3′<br>Reverse 5′-AGATCCCAGGAATTCAGCCCGAAG-3′	Sigma
FOSL1-SE	ChIRP-qPCR	Forward 5′-AATGAGGTCCCTGAGCCCACTG-3′<br>Reverse 5′-ACAGCAAGTCCCAGCAGAGTGG-3′	Sigma
SNAI2-SE	ChIP-qPCR	Forward 5′-GTGTGTTTTGTGGGAAATGGAGTG-3′<br>Reverse 5′-CTCTATGTGAGGGTTTGTTCGGA-3′	Sigma
SNAI2-SE	ChIRP-qPCR	Forward 5′-GGGCAGCAGAGCTGGTTTCCTA-3′<br>Reverse 5′-TCCTGAGAGCGTGGTCACTGGC-3′	Sigma
CYTOR promoter	ChIP-qPCR	Forward 5′-CTAGCTGTAGATGAAAACTGGGTC-3′<br>Reverse 5′-GTACCTGATGCTGTGTAGGCTAT-3′	Sigma
CYTOR	siRNA1	5′-CUAUGUGUCUUAAUCCCUU-3′	Ribobio
CYTOR	siRNA2	5′-GGUCUGGUCGGUUUCCCAU-3′	Ribobio
CYTOR	shRNA1	5′- GAAACAGGAAGCTCTATGA -3′	Sigma
CYTOR	shRNA2	5′- CCAGTCTCTATGTGTCTTA -3′	Sigma
FOSL1	siRNA1	5′-GCUCAUCGCAAGAGUAGCA-3′	Ribobio
FOSL1	siRNA2	5′-GAGCUGCAGUGGAUGGUAC-3′	Ribobio
